# Unraveling Multiphase Conversion Pathways in Lithium–Sulfur Batteries through Cryo Transmission Electron Microscopy and Machine Learning-Assisted Operando Neutron Scattering

**DOI:** 10.1021/acsnano.5c00536

**Published:** 2025-04-24

**Authors:** Jean-Marc von Mentlen, Ayça Senol Güngör, Thomas Demuth, Jürgen Belz, Milivoj Plodinec, Pronoy Dutta, Alen Vizintin, Lionel Porcar, Kerstin Volz, Vanessa Wood, Christian Prehal

**Affiliations:** † Department of Information Technology and Electrical Engineering, ETH Zürich, Gloriastrasse 35, Zürich 8092, Switzerland; ‡ Materials Science Center and Faculty of Physics, Philipps University Marburg, Hans-Meerweinstraße 6, Marburg 35043, Germany; § Department of Chemistry and Applied Biosciences, Scientific Center for Optical and Electron Microscopy, ETH-Zürich, Otto-Stern-Weg 3, Zürich 8093, Switzerland; ∥ Department of Chemistry and Physics of Materials, University of Salzburg, Jakob-Haringer-Straße 2a, Salzburg 5020, Austria; ⊥ Department of Materials Chemistry, National Institute of Chemistry, Hajdrihova 19, Ljubljana 1000, Slovenia; # Institut Laue–Langevin, 71 Avenue des Martyrs, Grenoble 38042, France

**Keywords:** lithium–sulfur batteries, cryogenic transmission electron microscopy, electron energy loss spectroscopy, small angle neutron scattering, machine learning

## Abstract

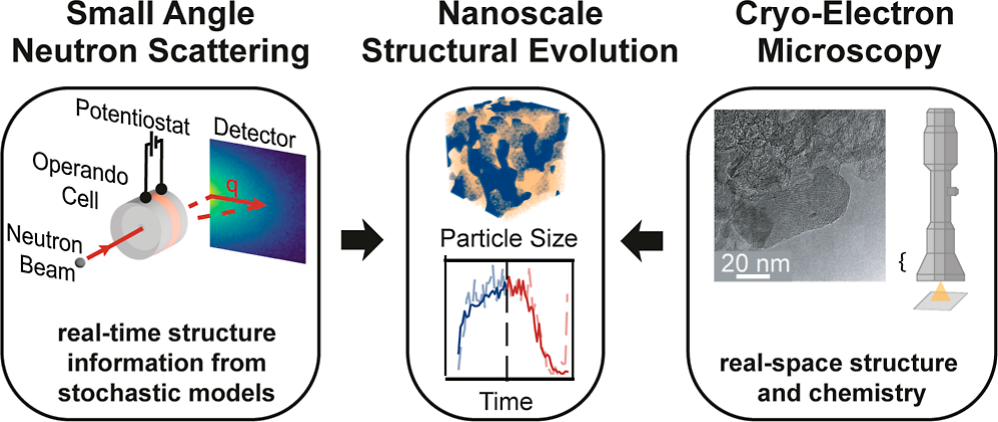

Understanding the complex physicochemical processes in conversion-type batteries requires investigations across multiple length scales. Here, we present a methodological approach to examine Li–S batteries on the nanoscale by combining cryogenic transmission electron microscopy (cryoTEM) with operando small-angle neutron scattering (SANS). CryoTEM revealed discharge products with a biphasic structure consisting of nanocrystalline Li_2_S within an amorphous Li_2_S_*x*_ matrix. Data analysis of complementary operando SANS measurements was accelerated by a convolutional neural network trained to predict scattering curves based on plurigaussian random fields, enabling comprehensive parameter space exploration for model fitting. Our findings are in line with disproportionation-driven deposition of Li_2_S_2_ particles that agglomerate and partially reduce to Li_2_S via solid-state conversion. This challenges the conventional view of direct, stepwise electroreduction of polysulfides at the electrode–electrolyte interface. Overall, our multitechnique approach demonstrates the value of combining localized high-resolution imaging with time-resolved operando scattering measurements to understand complex electrochemical conversion pathways in next-generation energy storage systems.

## Introduction

As the world transitions to renewable power production to mitigate the growing climate crisis, batteries emerge as pivotal for electric mobility and stationary energy storage. Emerging technologies beyond lithium-ion batteries promise enhanced energy density and reduced environmental impact from raw materials. Among these, conversion-type systems, including metal–air (Me–Air),^1^ metal–sulfur (Me–S) batteries,^2^ emerge as promising alternatives.

**Figure 1:**
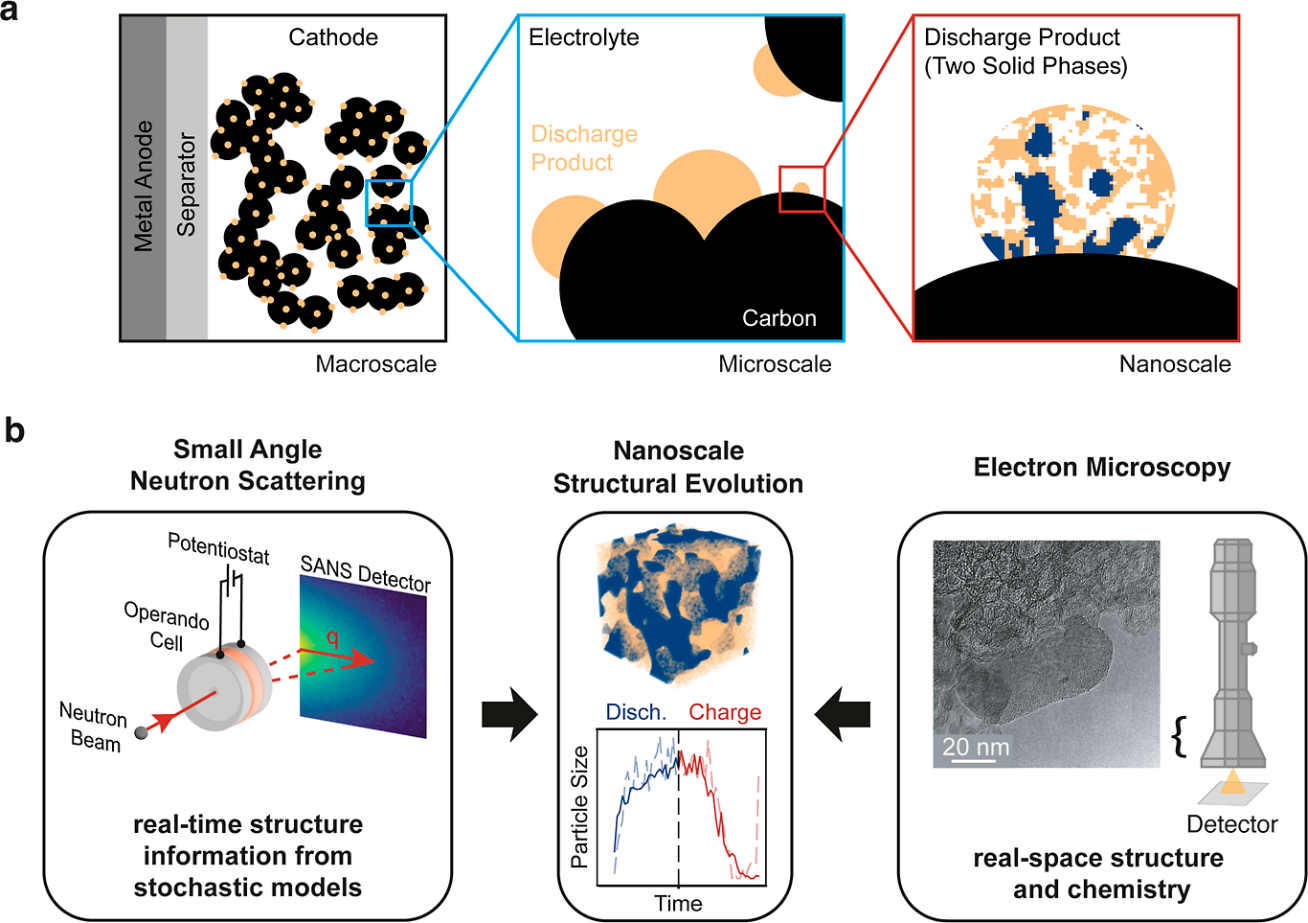
Multiscale analysis of deposition processes in conversion-type batteries using complementary techniques. (a) Schematic illustrating the hierarchical structure of a conversion-type cathode (here Li–S battery), from the macro-scale electrode to the nanoscale multiphase discharge product. (b) Complementary characterization methods: small angle neutron scattering (SANS) for operando structural evolution studies and electron microscopy for high-resolution imaging of the electrode morphology and composition.

Despite their potential, conversion-type batteries face common challenges that hinder their practical adoption. These challenges include poor ionic/electronic conductivity and charge transfer kinetics and large volume changes of active materials, as well as (partially) dissolved reaction products causing side reactions and suboptimal active material utilization, all of which constrain cycling rates, cycle life, and energy density.^1,3−5^ Further progress is limited by a lack of understanding of the complex structural transitions and physicochemical processes, particularly at the nanometer scale.^6−10^ The sulfur-to-sulfide conversion process in liquid-electrolyte lithium–sulfur (Li–S) batteries exemplifies these challenges. During discharge, crystalline sulfur (S_8_) is reduced to crystalline lithium sulfide (Li_2_S) through multiple soluble polysulfide intermediates (Li_2_S_8_, Li_2_S_6_, Li_2_S_4_, etc.). This solid–liquid–solid conversion process is critical for battery performance, as it dictates active material utilization and side reactions. However, the details of these processes remain unclear. Recent studies have shown that the complex nano- and microstructures of solid discharge products can consist of several phases (Figure 1a) and do not fit traditional nucleation and growth models, leaving many aspects of the conversion mechanism unresolved.^11−17^ To address this, advanced techniques are needed to probe sulfur-to-sulfide phase evolution at the atomic and nanometer scales.

Current techniques for studying conversion mechanisms at the atomic and nanoscale can be categorized into two groups: integral (or bulk) methods—including Raman, X-ray absorption spectroscopy, and both small- and wide-angle scattering—and local techniques, such as electron microscopy. Integral methods give insight into the ensemble-averaged structural and chemical composition of the electrode at a given time. Conducting such measurements operando can yield a detailed picture of the structural and chemical changes during cycling.^6,14−16,18−24^ In the case of scattering measurements, the data analysis relies heavily on the chosen model, leading to potential variations in the interpretations drawn from the same data set.^15,25,26^ Consequently, the process is susceptible to significant user bias, as the model selection can influence the conclusions reached. Microscopy techniques, on the other hand, offer model-free, localized chemical, and structural insights. However, their utility is constrained by a limited field of view and the requirement for specialized cell designs for in situ measurements.^27−32^ Additionally, the need for ultrahigh vacuum conditions and the electron beam-induced damage imposes significant limitations, particularly for materials like sulfur or lithium sulfide that are sensitive to such conditions.^33^

In this work, we present a methodological approach for investigating conversion-type batteries at the nanoscale, combining advanced microscopy and scattering techniques with machine learning-enhanced data analysis (Figure 1b). We demonstrate this approach using Li–S batteries as a prototypical system, leveraging the synergistic properties of transmission electron microscopy (TEM) and SANS. We investigate discharge products in Li–S battery cathodes by using ex situ energy filtered TEM (EFTEM) and electron energy loss spectroscopy (EELS) under cryogenic conditions to mitigate beam-induced damage. Air sensitivity is addressed using a vacuum-transfer cryo holder (VTC), and beam sensitivity is assessed under both noncryogenic and cryogenic conditions.^33,34^

To expedite the curve fitting process and allow for a broader parameter space exploration in SANS data analysis, we employ machine learning algorithms. We train a convolutional neural network (forwardCNN) to predict SANS intensity curves based on input parameters of the plurigaussian random field (PGRF) model, reducing computational time by 3 orders of magnitude. This significant speed-up enables the application of Bayesian optimization for efficient determination of optimal PGRF parameters.

Our results reveal that the discharge product in Li–S battery cathodes comprises multiple solid phases including nanocrystalline Li_2_S embedded in an amorphous polysulfide matrix. Real-time structural data indicate that these phases do not change their mean size simultaneously during cycling, offering new insights into the electrochemical conversion mechanisms. This study further showcases the potential of combining advanced imaging and scattering techniques with computational analysis to study a variety of battery chemistries with complex conversion mechanisms.

## Results and Discussion

### CryoTEM

**Figure 2:**
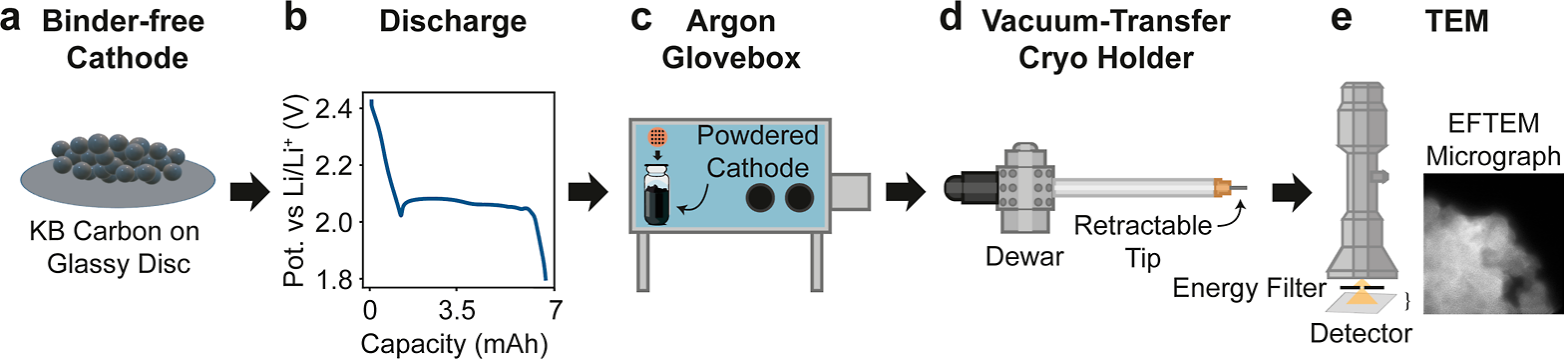
CryoTEM sample preparation and analysis workflow. A discharged cathode containing Ketjenblack carbon and Li_2_S_8_ catholyte is ground into a powder inside an Ar-filled glovebox. The sample is transferred to a TEM grid via direct immersion and loaded onto a vacuum-transfer cryo holder, which maintains inter conditions during transport to the microscope. In TEM, the sample is investigated using high-resolution imaging (HRTEM) and spectroscopic measurements like EFTEM and TEM-EELS.

Previous works investigating Li–S cathodes employed different strategies to mitigate beam- and low-pressure-induced changes to the sample. Most notable are encapsulations of sulfur in hollow carbon structures for solid-state Li–S batteries or the usage of liquid cell holders to measure electrochemical TEM.^13,27,28,32,35^ While these methods yield deep insight into the local processes during (de)lithiation, the carbons and cell geometry of such nanobatteries differ greatly from standard cells. Here, the ex situ TEM sample was prepared by discharging a catholyte solution of 0.5 M Li_2_S_8_ and 1 M LiTFSI + 0.4 M LiNO_3_ in diethylene glycol dimethyl ether (diglyme, 2G) onto a binder-free Ketjenblack (KB) powder (Figure 2a). The use of commercial high-surface-area carbon black KB ensured comparability between the investigated structures and the structures found in standard Li–S battery cathodes. The discharge curve revealed a distinct plateau at 2.1 V vs Li/Li^+^, indicating the formation of Li_2_S onto the KB powder (Figure 2b). After full discharge, the powder was vacuum-dried without washing, ground with a mortar, and then transferred onto a Lacey Carbon TEM grid under an inert argon atmosphere (Figure 2c). By omitting the washing steps, we avoid the risk of removing easily soluble compounds (such as higher-order polysulfides Li_2_S_*x*_, *x* > 1) and altering the structure of the discharge product.^14^Figure S4 shows the washing impact of different solvents on the structure. Next, the grid was loaded on the LN2 Vacuum Transfer Holder by MelBuild (Figure 2d) and transported under Ar atmosphere from the glovebox to the TEM (Figure 2e). The holder was then cooled to −193 °C by introducing liquid nitrogen (LN2) into the holder’s dewar. A more comprehensive description of the experimental procedure is provided in the Methods section.

**Figure 3:**
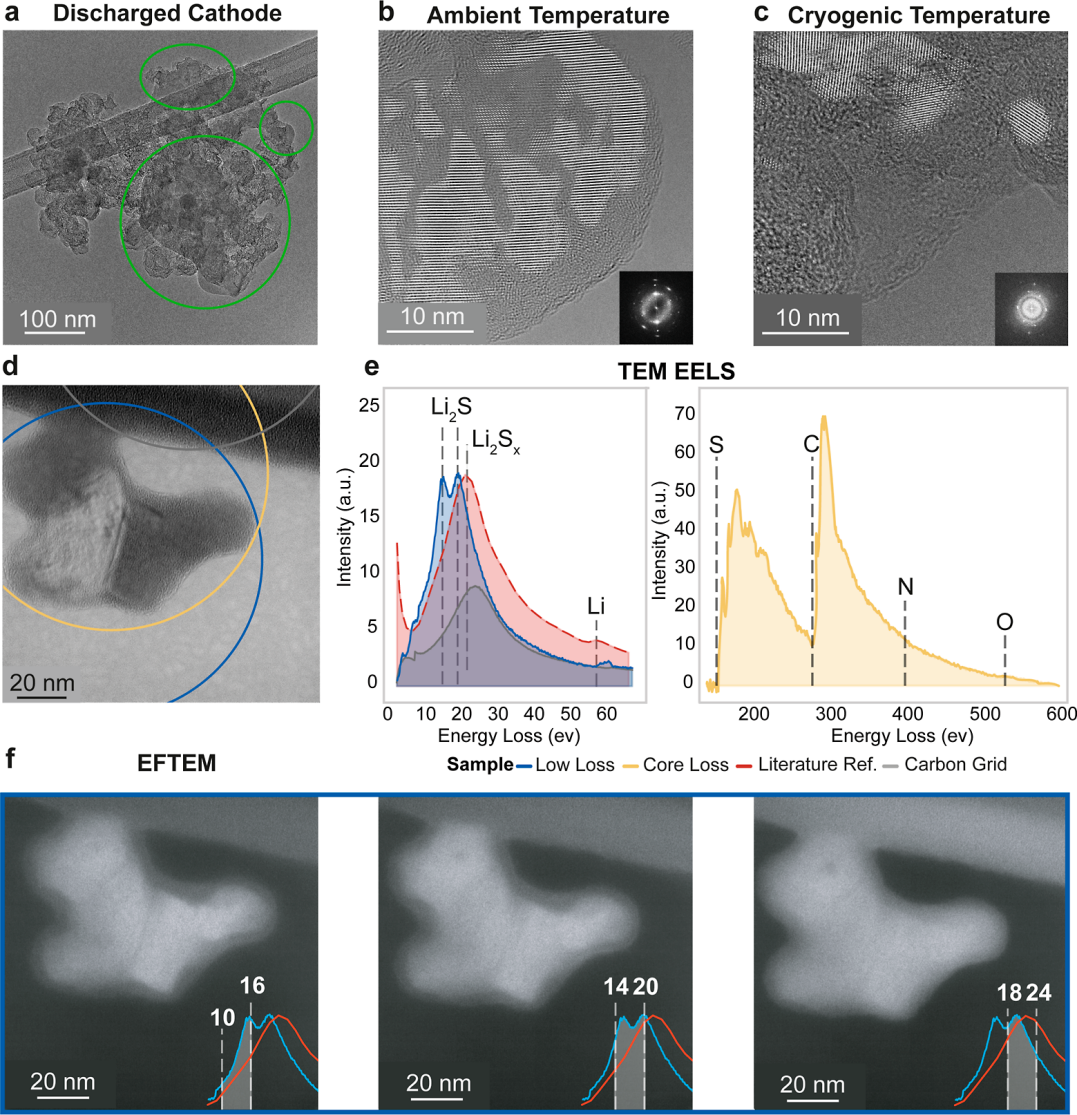
Multitechnique TEM analysis of discharged Li–S battery cathode. (a) Overview of a typical cathode region with discharge particles highlighted in green (cryoTEM). (b,c) Fourier filtered and magnified views of a two-phase discharge particle under ambient and cryogenic conditions, respectively. Insets show FFT spots assigned to (111), (200), (220), and (222) planes of Li_2_S crystals. (d) High-resolution image of a two-phase discharge product (Fourier filtered). The blue, yellow, and gray circles indicate the measurement area for the corresponding TEM-EELS spectrum in (e). The blue curve shows the characteristic Li_2_S double peak in the low-loss region. The sulfur edge onset (yellow) appears at 159 eV. Reference spectra for Li_2_S_*x*_ (red)^28^ and carbon structure (gray) are overlaid. (f) EFTEM series centered around the Li_2_S double-peak energies. The energy window for every micrograph is overlaid on the bottom right. Two distinct phases are visible at the double peak energies (13 ± 3 and 17 ± 3 eV), but the intensity difference diminishes at higher energy (21 ± 3 eV).

The measurements were conducted in TEM mode because of the low Z-contrast between sulfur and carbon in STEM mode (Figure S1b). Avoiding STEM further helped mitigate damage induced by the highly condensed electron beam. The mortared cathode particles on the TEM grid differ greatly in size. One such typical particle compromised of KB and discharge products is displayed in Figure 3a. The discharge particles range from a few dozen to hundreds of nanometers in size, consistent with previous SEM observations.^14,36^ We focused on discharge material at the edge of the larger particle to obtain an unobscured view of its structure. High-resolution micrographs of the discharge products, taken under both ambient and cryogenic conditions (Figures 3b,c and S1c–f), demonstrate that the samples remained stable regardless of temperature. The discharge products are made of two distinct solid phases: a nanocrystalline and an amorphous one. The nanocrystalline phase can be assigned to Li_2_S based on the fast Fourier transform (FFT) showing spots which can be assigned to the 111, 200, 220, and 222 planes (inset in Figure 3b,c, and in Figure S1 in large).

There are four candidates for the amorphous phase, based on the chemical composition of the battery: the salt LiTFSI, the solvent 2G, Li_2_S, or a higher order LiPS. The LiTFSI salt is visible throughout the sample, as the cathode was not washed. However, the micrometer-sized salt particles are clearly identifiable by their strong contrast and crystallinity (Figure S1f,g). Next, we turn to EELS measurements to gain a deeper understanding of the elements present. While the discharge product was stable under both ambient and cryogenic conditions for the HRTEM measurements, it degraded in under 1 s during cryo-STEM EELS measurements, likely due to the increased local e^–^-dose. We, therefore, turned toward cryoTEM-EELS and cryo-EFTEM, sacrificing the local resolution of the elemental distribution. The e-dose were roughly 200 e/Å^2^ for EFTEM and up to 267 e/Å^2^ for STEM per frame. The discharge product in Figure 3d was measured at different energy levels and locations. The circles indicate the field-of-view during the TEM-EELS measurement, and their colors relate to the low- and core-loss spectra shown in Figure 3e. The low-loss spectrum in blue shows a distinctive double peak plasmon feature (15.5 and 19.7 eV) that can be attributed to Li_2_S.^27,28^ The red curve shows a reference spectrum of Li_2_S_*x*_ taken from Yang et al.^28^ The core-loss spectrum (yellow), acquired from the discharge product and the supporting carbon structure, reveals two significant edges: sulfur (159 eV) and carbon (284 eV). Notably, the sulfur edge appears at lower energy compared to bulk sulfur (165 eV), consistent with previous reports for Li_2_S.^27,28,32^ The absence of nitrogen (402 eV) and oxygen (532 eV) edges further suggests that the amorphous phase consists of other amorphous Li_2_S or an amorphous solid Li_2_S_*x*_ (LiPS) phase. LiTFSI and residual 2G, both rich in oxygen and nitrogen, can be excluded as constituents of the amorphous phase.

While EELS can typically differentiate between Li_2_S and polysulfides, particularly in the low-loss region,^28^ our lack of STEM’s local resolution prevents direct distinction without sample alteration. To address this limitation, we acquired a series of EFTEM micrographs with energies filtered at around 13, 17, and 21 eV (Figure 3f). Insets in the micrographs show a detailed view of the low-loss EELS spectra of Li_2_S and Li_2_S_*x*_. The low-loss spectrum remains unaltered after isolating and subtracting the zero-loss peak (Figure S2a,b), which suggests the sample to be sufficiently thin for any brightness differences to arise from chemical composition rather than thickness variations. The first two micrographs, centered around the Li_2_S peaks (13 and 17 eV), reveal two distinct brightness regions: a brighter phase in the inner region and a darker phase in the outer areas. At higher energies (21 eV), this brightness difference disappears, making the particle appear as a single phase. These findings indicate that the amorphous phase covering the particle must be solid Li_2_S_*x*_. Consequently, the solid discharge product in Li–S batteries comprises nanocrystalline Li_2_S embedded within amorphous Li_2_S_*x*_, which is in line with our previous study combining Raman spectroscopy with neutron and X-ray scattering.^14^

### Operando SANS

**Figure 4:**
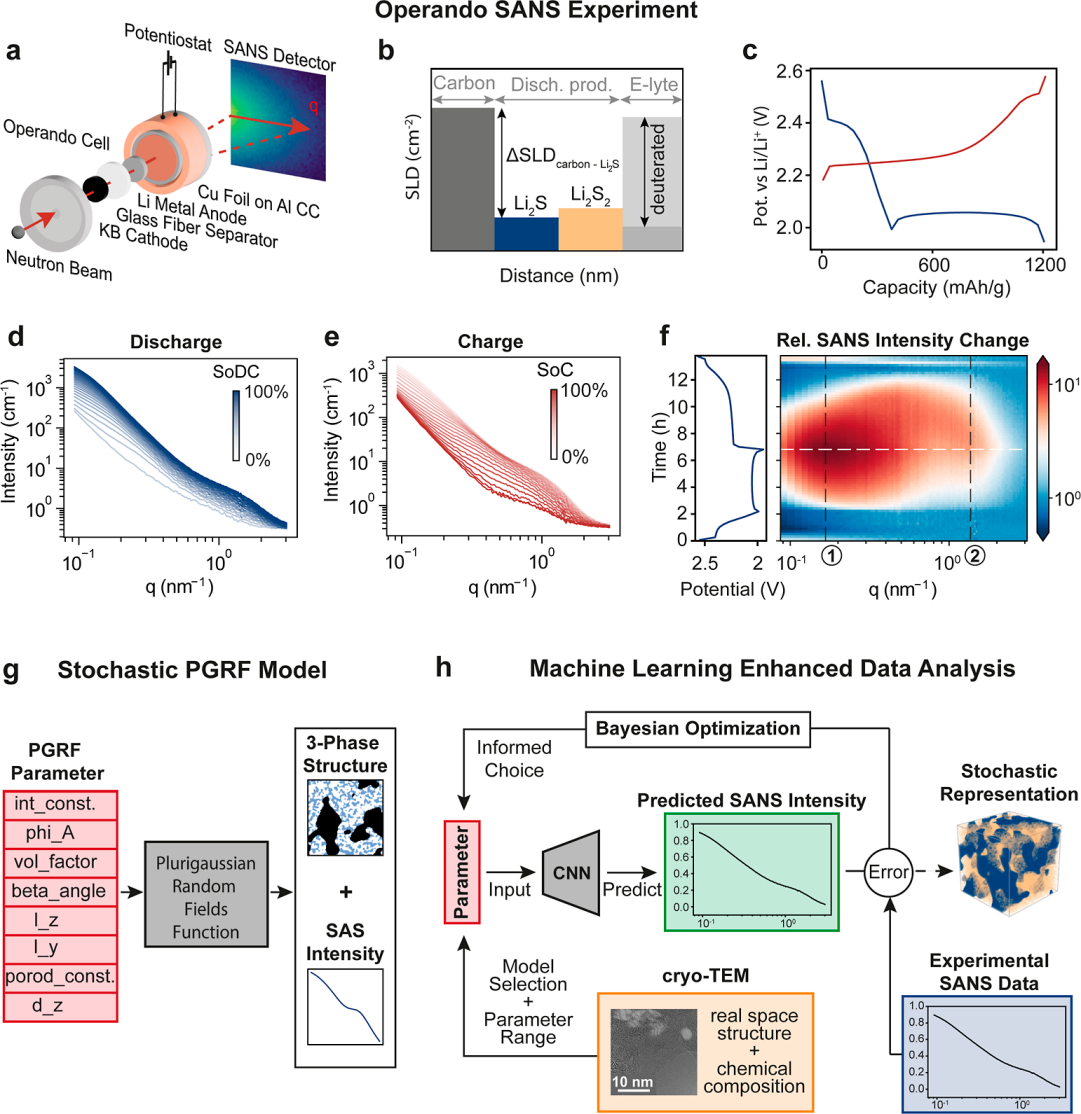
Operando SANS results and machine-learning supported data analysis pipeline. (a) Sketch of SANS cell configuration and experimental setup at ILL. (b) Differences in scattering length densities (SLD) visualized for the different materials. The SLD of the electrolyte was matched to the carbon using a deuterated solvent. (c) (Dis-)charge profile of operando cell. The discharge and charge cycles are color-coded blue and red, respectively. (d) Integrated intensity curve acquired during the discharge cycle and (e) charge cycle, with color opacity indicating the state of (dis-)charge (SoDC, SOC). (f) The two intensity shoulders are highlighted by plotting the relative intensity and marked as (1) for low-*q* and (2) for high-*q*. (g) The PGRFs function takes in 10 structural parameters and outputs a 3-phased stochastic structure with the corresponding small angle scattering intensity curve. (h) CryoTEM data guide initial model selection and help in constraining initial PGRF parameter ranges and model selection. A pretrained CNN predicts SANS intensity curves based on input parameters, which are then compared to experimental data. A Bayesian optimization algorithm refines parameter selection based on the error, iterating until convergence or a set iteration number. Final PGRF parameters for each state of charge are used to generate stochastic 3D representations of the electrode structure. This workflow combines structural insights from cryoTEM with machine learning techniques to efficiently analyze SANS data and reconstruct the evolving electrode nanostructure.

Next, we turn toward operando SANS to further understand the biphasic discharge product and its behavior during galvanostatic cycling. For operando SANS measurements, we installed a custom-built operando SANS cell at the D22 beamline at ILL Grenoble (Figure 4a).^14,24^ A neutron beam of about 10 mm in diameter hit the Li metal, separator, and the Li–S battery cathode. Control experiments ensured that we observed changes in the Li–S battery cathode only.^14^ We used battery chemistry equivalent to the one we used for cryoTEM measurements, using a free-standing sulfur infiltrated KB composite electrode (KB/S) with PTFE binder and 1 M LiTFSI + 0.4 M LiNO_3_ in 2G as electrolyte. The 2G solvent is deuterated to achieve a scattering length density (SLD_el_ = 5.63 × 10^–10^ cm^–2^) that approximately matches the carbon’s scattering length density (SLD_C_ = 6.67·× 10^–10^ cm^–2^) with a slight mismatch of 15.6%. The square of the SLD difference between active materials and both electrolytes/carbons is about 30 times higher than the square of the carbon-electrolyte SLD difference. This ensures that any variations in the SANS curves are predominantly due to changes in the nanostructure of the discharge products, as depicted in the sketch in Figure 4b.

The galvanostatic dis-/charge curves, shown in Figure 4c, reveal the standard features of Li–S batteries with ether-based electrolytes: (i) a high voltage plateau around 2.3–2.4 V corresponding to the dissolution of sulfur and its electrochemical reduction into polysulfides (Li_2_S_8_), followed by (ii) a second plateau above 2.0 V, which marks the coexistence of dissolved polysulfides and solid discharge products like Li_2_S and (iii) a charging plateau above 2.2 V vs Li/Li^+^.^37^ Prior to discharge, the SANS curve reflects the constant contribution from the separator, PTFE binder, and carbon black due to imperfect SLD matching between the deuterated electrolyte and carbon. The constant background at higher scattering vector *q* originates from incoherent scattering and the electrolyte, carbon structure, and sulfur structure factor. During discharge, the overall SANS intensity increases and two distinct features (intensity shoulders) emerge at low and high *q*, stemming from the nanostructure of the solid discharge products (Figure 4d). During charging, the SANS intensity decreased again (Figure 4e). The contour plot in Figure 4f reveals the relative intensity change (normalized by the SANS intensity prior to discharge) as a function of time and *q*; the shoulders in Figure 3d,e appear as intensity maxima. The intensity increase, indicative of solid discharge product formation, starts with the onset of the lower voltage plateau. During charging, the overall intensity decreases, and the high-*q* intensity maximum shifts toward lower *q*-values. The positions of low-*q* and high-*q* intensity shoulders at around (1) 0.2 and (2) 1.5 nm^–1^ suggest structures with feature sizes of around 2π/0.2 nm^–1^ ≈ 30 nm and 2π/1.5 nm^–1^ ≈ 4 nm, respectively. During charging, the high-*q* shoulder shifts slightly toward the lower *q*, and both features decrease in intensity until they vanish completely (Figure 4e). A similar behavior was previously reported under different experimental settings with small-angle neutron and X-ray scattering.^14,19^

To identify the origin of the high-*q* intensity shoulder, we performed small- and wide-angle X-ray scattering (SAXS/WAXS) measurements on discharged KB/S electrodes (Figure S3). The WAXS data revealed the expected diffraction peaks of nanocrystalline Li_2_S (peaks (111) and (200)) in the discharged electrode. After washing the discharged electrode with diglyme, the Li_2_S diffraction peak remained, while the high-*q* SAXS feature disappeared, unlike in the unwashed sample. This suggests that the high-*q* feature observed in the SANS data corresponds to the soluble Li_2_S_*x*_ phase seen in TEM. Based on our SAXS/WAXS analysis, previous Raman spectroscopy results, and other theoretical and experimental studies, we propose that this Li_2_S_*x*_ phase is an amorphous, particulate Li_2_S_2_ phase.^12−14,38^ Hence, the low-*q* and high-*q* intensity shoulders observed in Figure 4d–f, along with the cryoTEM results, indicate that the nanostructure comprises larger Li_2_S aggregates (around 30 nm, associated with the low-*q* shoulder) and an amorphous nanoparticulate Li_2_S_2_ phase (feature size of around 4 nm, associated with the high-*q* shoulder). These Li_2_S aggregates consist of individual crystallites with an average size of 14 nm, as confirmed by both Scherrer analysis (Figure S3c) and direct TEM observation (Figure 3b,c). This structural composition is the base assumption for the following operando SANS data modeling approach.

### Machine Learning for the PGRF/SANS Model

CryoTEM revealed that the discharge product consists of at least two solid phases. To extract the nanoscale structural evolution of the two-phase solid discharge products from the operando SANS experiment, we chose the PGRF model.^14,39,40^ This stochastic structural model effectively generates a small-angle scattering curve and a statistically representative real-space structure composed of three distinct phases: nanocrystalline Li_2_S, the surrounding amorphous particulate Li_2_S_2_ phase, and the electrolyte (Figure 4g).

The PGRF model employs 13 input variables: 10 structural parameters that define the structure geometry and 3 material parameters representing the SLDs of the constituent materials. These inputs are used to calculate the corresponding scattering curve, effectively creating a Fourier transform of the SLD correlation function of the real-space physical structure. More details are explained in our recent study, in the Methods section and in Gommes et al.^40^Figure S5 visualizes the concept. We implemented the functions in Python 3, accessible on GitHub.^41^ In this performance-optimized version, the calculation of a single scattering curve takes approximately 2.4 s on our system. While this might seem fast, applying a curve fit to our measurement data with a widely unrestricted parameter space would still take several months.

To overcome this computational challenge, we turned to machine learning (Figure 4h). We trained a convolutional neural network, which we call forwardCNN, to predict the SANS curve based on the input parameters of the PGRF function. Choosing the right structural stochastic model and parameter range requires apriori information about the expected nanostructure from cryoTEM measurements. The cryoTEM results indicated two solid discharge products in the nanometer range, for which the PGRF model is ideal. The experimental SANS intensities are then fitted by minimizing the error between the CNN-predicted SANS intensity and the experimental SANS intensity by using Bayesian optimization. Once the PGRF parameters with a minimum error are found, a stochastically representative real-space model can be calculated.

Convolutional neural networks, commonly used in image recognition, are particularly effective at identifying patterns in complex data.^42^ In our case, the network learns to recognize how changes in the PGRF parameters affect the resulting scattering curve. For training, we generated 250,000 SANS intensity/input parameters sets using the PGRF model (Figure 4g). The input parameters for the PGRF model varied over a broad range, covering any realistic three-phase nanostructure with feature sizes from 0.5 to about 50 nm (for details, see the Methods and Supporting Information). The base structure and training results of our forwardCNN are shown in Figures S8–S15. The model achieves high precision, with an average mean squared error <10^–5^ on the intensity-normalized data set, while taking only 25 ms per prediction. This represents a speed-up of 3 orders of magnitude compared to the traditional calculation method. A more detailed performance analysis as well as an alternative model (inverseCNN) that predicts the PGRF parameters directly from the SAS curve is given in the Supporting Information. Combining the fowardCNN with Bayesian optimization is particularly advantageous: the forwardCNN provides flexibility, accurately predicting SANS curves even when some input (fitting) parameters are held constant, while Bayesian optimization excels at navigating the complex, nonconvex parameter space of the PGRF function. The result is a powerful, machine learning-enhanced optimization loop that can fit a single curve in about 4 min, testing 10,000 different parameter combinations. This speed and efficiency open up new possibilities for real-time analysis and interpretation of SANS data during experiments and offer new opportunities to study dynamic processes in battery materials.

### Fitting Results

**Figure 5:**
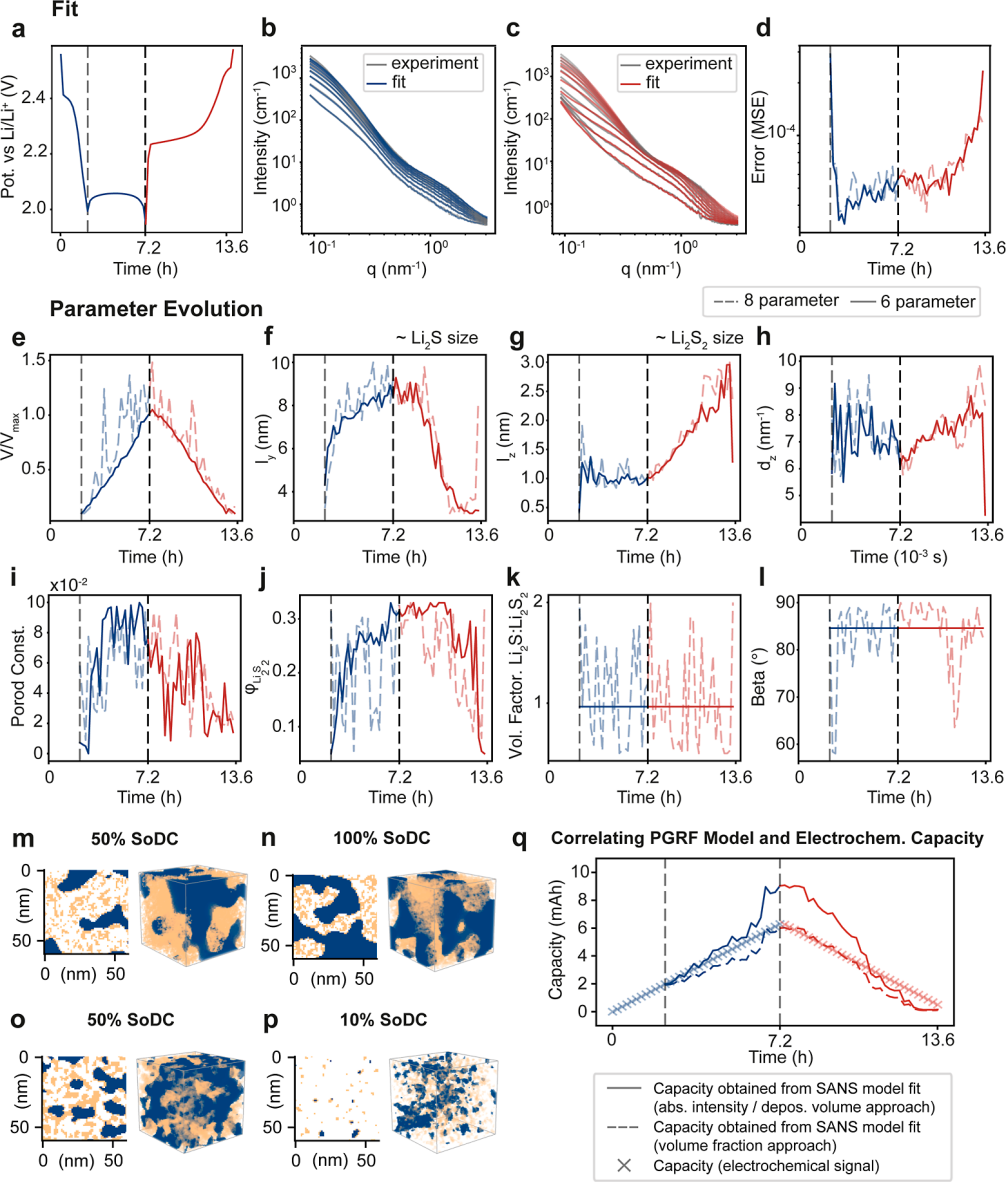
Evolution of PGRF parameters over a full discharge–charge cycle. Results from two fitting rounds are shown: one with 8 varying parameters (dashed lines) and another with 6 (solid lines). (a) Discharge profile color-coded for the discharge (blue) and charge (red) cycle. (b,c) Experimental SANS data (gray) with fitted curves overlaid in color. (d) Corresponding fit error values. (e) Intensity constant, indicating the amount of deposited material. (f,g) Particle size evolution of Li_2_S and Li_2_S_2_, respectively. (h,i) Porod constant and d_*z*_ parameter, showing visible trends over the cycle. (j) Volume fraction of Li_2_S_2_ in the discharge product. (k,l) β angle and the volume factor oscillated around constant values and were fixed in the second fitting iteration. (m–p) PGRF cross section and volumes for a given state of charge generated using the fitted parameters. (q) Correlation between capacity measured electrochemically (crosses) and capacity derived from the PGRF model using two different approaches: absolute intensity/deposition volume (solid line) and volume fraction (dashed line).

Figure 5 illustrates the experimental and fitting processes (a–d) and the retrieved parameter sets for every curve (e–l). The electrochemical cycle in Figure 5a is divided into discharging (blue) and charging (red) segments. The first plateau at 2.4 V corresponds to the dissolution and conversion of sulfur to higher-order polysulfides (Li_2_S_*x*_, 6 ≤ *x* ≤ 8). Solid discharge products, consisting of Li_2_S_2_ and Li_2_S, are formed at the second plateau around 2.1 V. As the SANS experiment is sensitive only to the solid discharge products, we fit the scattering curves starting with the onset of the second plateau. The fitted curves in Figure 5b,c (blue and red) closely match the operando SANS curves (gray). This strong agreement is further supported by the normalized mean square error shown in Figure 5d. The error increases significantly at the beginning and end of the cycle, which is when signal intensity and contrast are lowest due to the absence of discharge products.

To gain deeper insights into the structural evolution during cycling, we examined the PGRF parameters obtained from each fit, as shown in the bottom panels of Figure 5e–l. We preselected eight out of the ten structural parameters based on a parameter study shown in Figure S6, which demonstrate the impact of each parameter on the structure. To explore the robustness and flexibility of our approach, we conducted two fitting rounds. In the first round, all eight preselected parameters were allowed to vary freely. The results revealed that only six parameters in Figure 5e–j showed a time-dependent trend, while the other two in Figure 5k–l oscillated around constant values. In a second fitting round, the two oscillating parameters were fixed at their mean values, which significantly reduced the noise in the remaining parameters without substantially affecting the overall fit quality. As highlighted by the parameter study in Figure S6, changes in the volume ratio (Figure 5l) appear to have correlated effects on the SANS intensity like the parameters *V*/*V*_max_ and φ_Li_2_S_2__. When the volume ratio was allowed to vary, these parameters showed increased noise, suggesting an overextension of the Bayesian fitting algorithm. This interpretation is further supported by the inverse CNN model (Figure S15), which similarly struggled to accurately predict the volume ratio from the scattering curves. Overall, this two-step fitting process demonstrates the flexibility of the model fit to any number of fitting parameters, while the trained CNN remains the same. It allows for a systematic fitting procedure and identifies strongly correlated fitting parameters or parameter degeneracy.

Analyzing the key parameter trends in Figure 5e–g reveals important insights. The normalized volume of solid discharge products increases approximately linearly with time during discharge and decreases linearly with time during charge (Figure 5e). Notably, while the Li_2_S aggregate size (approximately 2.5 times *l*_*Y*_ in Figure 5g) increases during discharge and decreases during charge, the Li_2_S_2_ particle size (approximately 2.5 times *l*_*Z*_ in Figure 5h) remains constant during discharge and increases during charging.^14^ This observation aligns with the *q*-shift of the high-*q* intensity shoulder in Figure 4f, indicating Li_2_S_2_ structural evolution. As the volume factor parameter in Figure 5l was held constant, the volume ratio between Li_2_S and Li_2_S_2_ was also fixed in our model. However, the overall volume fraction of Li_2_S and Li_2_S_2_ with respect to the surrounding pore space changes slightly, as shown in Figure 5j. Figure 5m–p reveals the statistically representative real-space structures of the discharge product, calculated from the PGRF approach with the corresponding fitting parameter results at 50% discharged, 100% discharged, 50% charged, and 90% charged states, as an input. These 3D real-space cross sections visualize the significant structural evolution of the Li_2_S and Li_2_S_2_ phases throughout the cycling process and reflect the parameter changes discussed above.

Our structural analysis reveals a complex conversion process involving metastable intermediate phases, such as solid Li_2_S_2_, which can persist even at the end of discharge. Therefore, explanations of galvanostatic discharge/charge profiles based solely on equilibrium phase diagrams^37^ may be insufficient. Considering the kinetics and metastable phases seems necessary to fully grasp electrochemical sulfur conversion.

We further compared our results from the SANS model fit with experimental electrochemical data. In particular, we calculated capacities from the found Li_2_S and Li_2_S_2_ fractions and compared them to capacities obtained from the electrochemical signal (Figure 5q). The dashed line represents the capacity evolution assuming complete conversion of sulfur to Li_2_S and Li_2_S_2_ and taking the found Li_2_S/Li_2_S_2_ volume fractions (φ_Li_2_S_2__ and φ_Li_2_S_) as an input. This SANS model result closely matches the electrochemical capacity at 100% state of discharge (SoDC, mismatch <1%). The capacity values calculated from the electrode’s deposit volume *V* (derived from *V*/*V*_max_ in Figure 5q) multiplied by the Li_2_S_2_ and Li_2_S volume fractions (φ_Li_2_S_2__ and φ_Li_2_S_) exceed the experimental value by approximately 35% (solid line in Figure 5m). Possible sources of error include (i) a slight error in the absolute intensity calibration, (ii) a minor deviation from the nominal electrode diameter (e.g., a few percent deviation from 13 mm could significantly impact calculations), or (iii) inhomogeneous Li_2_S/Li_2_S_2_ deposition across the electrode.

### A Holistic Data Interpretation

**Figure 6:**
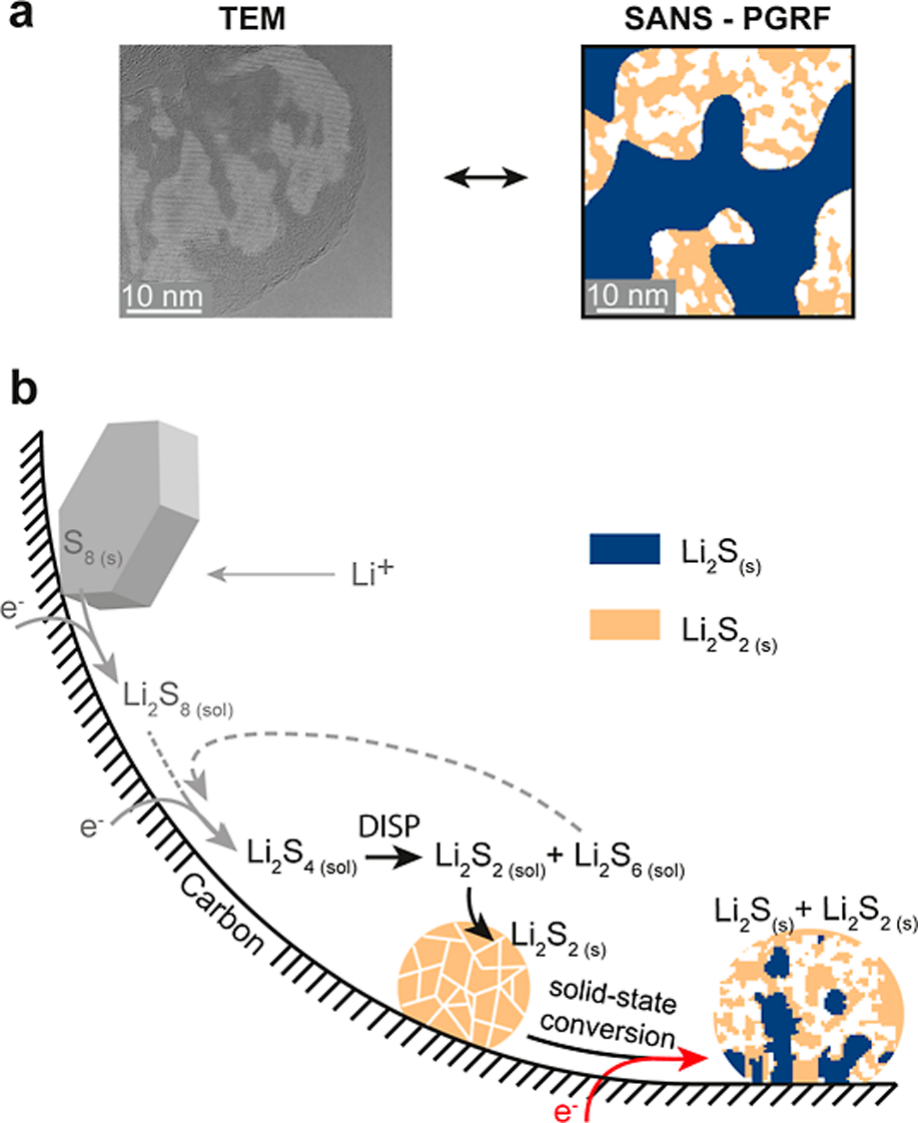
Proposed formation mechanism of Li_2_S/Li_2_S_2_ in nonaqueous lithium–sulfur batteries. (a) Comparison of composite solid discharge products obtained with TEM and PGRF modeling/SANS. (b) The schematic illustrates the stepwise reduction of polysulfides during discharge. Dissolved Li_2_S_8_ is initially reduced to shorter-chain polysulfides (Li_2_S_*x*_ 4 ≤ *x* ≤ 7) near the carbon surface. Solid Li_2_S_2_ aggregates precipitate from solution, likely through disproportionation (DISP) of Li_2_S_4_. The Li_2_S_2_ particles subsequently undergo solid-state conversion to form Li_2_S crystals. Such conversion would preferentially occur near the triple-phase boundary of carbon, electrolyte, and Li_2_S_2_, resulting in a gradient of Li_2_S fraction within the discharge product.

It is crucial to merge the complementary information obtained from cryoTEM and operando SANS results (Figure 6a). By relating the fitted parameters from operando SANS to electrochemical data and the phase distribution observed in cryoTEM, we can build a comprehensive understanding of the sulfur conversion mechanism. CryoTEM reveals two key observations: (1) Li_2_S forms crystallites up to 10 nm in size, which aggregate into larger structures and (2) these Li_2_S aggregates are embedded in an amorphous Li_2_S_*x*_ phase, which is likely Li_2_S_2_. This aligns with SANS model results, indicating a composite structure of Li_2_S aggregates (of approximately 30 nm in size) and an amorphous nanostructured Li_2_S_2_ phase (with feature sizes up to 4 nm). The evolution of these phases during discharge and charge is crucial to understanding the reaction mechanism. The volume fraction of solid discharge products shows a positive correlation with capacity during discharge as phases are formed and decreases during charge as they dissolve (Figure 5e). While the Li_2_S aggregate size steadily increases during discharge and decreases during charge, the Li_2_S_2_ feature size remains constant during discharge and increases during charge (Figure 5f,g). Our findings align with computational studies predicting a stable solid phase of Li_2_S_2_ and its favorable formation pathway from Li_2_S_4_.^43,44^ A recent in situ TEM study has provided direct evidence for transient Li_2_S_2_ and amorphous lithium polysulfide deposits, further supporting our outcomes.^13,38^

Based on these insights, we propose a sulfur-to-sulfide conversion mechanism, as illustrated in Figure 6b. Crystalline sulfur is reduced to dissolved polysulfides such as Li_2_S_8_ or Li_2_S_6_, which are further reduced to Li_2_S_4_. As seen by X-ray absorption and Raman spectroscopy, dissolved Li_2_S_4_ tends to disproportionate into Li_2_S_6_ and Li_2_S_2_ in ether-based electrolytes,^14,45^ leading to the formation of larger Li_2_S_2_ aggregates via precipitation from solution. This process aligns with two key experimental observations: (1) the size of the larger aggregates is significantly influenced by the applied current or overpotential, which can be explained by classical nucleation and growth mechanisms;^36,46,47^ (2) the feature size of the Li_2_S_2_ nanostructure (approximately 4 nm) remains the same across various systems and experimental conditions, likely due to local transport limitations during the disproportionation reaction.^14,15,19^ Once the Li_2_S_2_ nanostructure is formed, it is partially reduced to Li_2_S through electroreduction. We hypothesize that mass transport for this solid-state reaction occurs via interfaces in the Li_2_S_2_ nanostructure. Given that electroreduction requires a direct pathway between the carbon host and the formed Li_2_S, we believe that the prevalence of the amorphous phase increases with the distance from the carbon surface. While some of our TEM data suggest that more Li_2_S is found in close proximity to the carbon host (Figure S1), a more in-depth analysis is required to confirm this mechanism. This fact could explain why Li–S batteries cannot reach their theoretical capacity.

## Conclusions

In conclusion, this study establishes a comprehensive experimental framework for investigating the electrochemical phase transformation in conversion-type batteries, as exemplified by lithium–sulfur batteries. By integrating cryoTEM/EELS for high-resolution structural and chemical exploration with machine learning-enhanced operando SANS for real-time statistical nanostructure analysis, we have developed an effective approach to understand electrochemical processes at the nanoscale.

Our cryoTEM and EELS investigations provided structural and chemical data, laying the groundwork for subsequent model selection and adaptation in operando SANS studies. The implementation of our forwardCNN significantly reduced the computation time of the SANS model fit based on stochastic modeling. This allowed us to explore a wide range of parameter spaces and generate statistically representative real-space structures of complex three-phase nanostructures. By reducing the need for parameter constraints during the model fit, this method decreases user-imposed restrictions and enables a detailed, quantitative analysis of nanostructure evolution during battery cycling.

A key finding of our methodology is the identification of a Li_2_S/Li_2_S_*x*_ biphasic discharge product in Li–S batteries. The consistency between cryoTEM and operando SANS results give evidence for a two-step mechanism involving Li_2_S_2_ precipitation from solution followed by solid-state electrochemical reduction to Li_2_S. This mechanistic insight highlights the significance of real-time tracking of nanoscale phase evolution, allowing for a deeper understanding of the physicochemical mechanisms governing complex conversion-type battery systems.

The approach presented here not only elucidates the lithiation mechanism in Li–S batteries but also offers a versatile framework that can be applied to a broad range of conversion-type battery chemistries and beyond. Our work underlines that next to thermodynamic equilibrium states^37^ also the path to equilibrium, often complicated by metastable intermediate states, is crucial for understanding the properties of conversion-type battery systems.^37^

## Methods

### Materials

For operando SANS measurements, we used carbon/sulfur composite cathodes with a C/S mass-ratio of 1:1. The carbon was a carbon black material (Ketjenblack EC-600JD, sourced from ANR Technologies) with a Brunauer–Emmett–Teller (BET) specific surface area of 1400 m^2^ g^–1^ and minimal metal contamination (<30 ppm). For melt infiltration, we first mixed the carbon with elemental sulfur at the mass ratio of 1:1, manually with pestle and mortar for about 5 min. The prepared mixture was then melt-infiltrated at 155 °C for 7–8 h in a sealed evacuated glass oven (Büchi, Switzerland). The final sulfur mass content was verified by weight.

To create free-standing electrodes, we combined the carbon/S composite with polytetrafluoroethylene (PTFE, acquired as a 60% aqueous suspension from Sigma-Aldrich) in a 90:10 mass ratio, using 2-propanol (≥99.8%, Sigma-Aldrich) as a dispersant. The components were manually blended in a mortar for 10 min at room temperature (25 °C). We then rolled the resulting paste into films of 180 ± 10 μm thickness. These films underwent a cleaning process using a mixture of acetone (≥99.5%, Sigma-Aldrich) and deionized water (18 MΩ cm), followed by overnight vacuum drying at 120 °C and 10 mbar pressure.

The deuterated electrolyte consisted of 1 M lithium bis(trifluoromethane)sulfonimide (LiTFSI, 99.95% trace metals basis) and 0.4 M lithium nitrate (LiNO_3_, 99.99% trace metals basis) dissolved in 100% deuterated diethylene glycol dimethyl ether (2G, anhydrous, 99.5%) from Cambridge Isotope Laboratories Inc.

The catholyte used for TEM investigations consisted of 0.5 M Li_2_S_8_, 1 M LiTFSI (99.95% trace metals basis), and 0.4 M LiNO_3_, 99.99% trace metals basis, dissolved in 2G (anhydrous, 99.5%).

For the electron microscopy sample preparation, we fabricated a binder-free cathode. To achieve that, we created a thick paste by mixing the carbon black powder with the catholyte at a ratio of 20 mg/50 μL. This paste was applied to a glassy carbon disc (SIGRADUR G Discs, 16 mm diameter, 0.5 mm thickness) and assembled in in-house-made coin-cell-type cells (uniaxial pressure of 0.7 ± 0.1 MPa), utilizing a polyethylene (PE) separator (Targray, PE16A) and Whatman separator. We added 100 μL of extra catholyte to ensure proper wetting of the separators. The electrolyte-to-sulfur ratio (E/S) and electrolyte-to-carbon ratio (E/C) were 7.8 and 7.5, respectively.

To synthesize Li_2_S_8_, we combined stoichiometric amounts of elemental sulfur (powder, 99.98% trace metal basis, Sigma-Aldrich) and lithium metal (110 μm thick high-purity foil, FMC Lithium Corporation) in excess anhydrous tetrahydrofuran (THF, ≥99.9%, inhibitor-free, Sigma-Aldrich). The THF underwent a multistep drying process using Al_2_O_3_ and molecular sieves, followed by distillation. We verified the water content using Karl Fischer titration (Mettler Toledo C20), ensuring it remained below 2 ppm. The entire synthesis took place in an argon-filled glovebox with strictly controlled atmosphere (H_2_O and O_2_ < 0.1 ppm). The mixture was heated to 50 °C and stirred until complete dissolution occurred. Finally, we removed the THF under vacuum (10 mbar) to obtain the dry polysulfide powders.

### Experimental Methods

Experimental operando SANS experiments were conducted under galvanostatic discharge/charge conditions at the ILL’s D-22 beamline, utilizing a wavelength of 0.5 nm and a 10 mm beam diameter.^48^ The setup included two areal detectors positioned at distances of 17.6 and 1.4 m for an overlapping *q*-region. The custom two-electrode SANS cell^14^ consisted of a PEEK body and two aluminum elements contacting the cathode and anode from the top and the bottom. Twelve mm aluminum windows ensures enough transmission for incident and scattered neutrons and uniform mechanical pressure. The cell stack comprised copper (≥99.9%, Schlenk Metallfolien) and aluminum foil current collectors (≥99.5%, Korf), a lithium metal anode (≥99.9%, Alfa Aesar, 0.75 mm thickness, 16 mm diameter), a glass fiber separator (Whatman GF/A, 21 mm diameter), and a KB/S cathode with 13 mm in diameter, prepared according to the procedure above. The operando cell, with E/S and E/C values of 38.3 and 31.3, respectively, was discharged at C/10. The neutron beam hit all cell components, with only the cathode showing reversible and notable structural changes. The 2D detector signals were azimuthally averaged and corrected for empty cell scattering and the detector dark current. Finally, the SANS intensities were normalized to absolute intensities (in cm^–1^) using transmission correction, detector efficiency correction, D22 detector parallax correction, and by dividing the data by the sample volume (assuming a sample thickness of 0.007 cm) and empty beam flux.

The ex situ SAXS/WAXS measurements (Figure S3) of the discharged KB/S cathode (galvanostatic discharge at C/10) were carried out at a laboratory SAXS/WAXS system (Xeuss 3.0 HR, Xenocs) using a copper microsource (with CuKα radiation), a 2D areal SAXS detector (Eiger 2R 1M, Dectris), and a 2D areal WAXS detector (Eiger 2R 500 K, Dectris).

For TEM measurements, the cathode was prepared as described above. Next, the cell was discharged with a C/20. After discharge, the cell was disassembled and the KB was scratched off the GC disc. The KB flakes were subsequently dried under a vacuum and ground into a fine powder using a mortar. This sample was then transferred to a Lacey Carbon Type-A Copper TEM grid (TedPella, no. 01890) by directly immersing the grid into the powder.

The different cycling rates (C/10 for SANS, C/20 for TEM) were chosen based on the requirements of each technique. The slower rate for TEM samples optimized the preparation of binder-free cathodes, ensuring a better sample transfer and higher discharge capacity. Similarly, for cryoTEM experiments, the Li_2_S_8_ catholyte was used instead of sulfur-infiltrated cathodes to ensure precise control of the active material amount and enable good electronic contact in the binder-free cathodes, which was essential for proper sample transfer onto the TEM grid. This difference in starting materials does not affect the mechanistic interpretations, as elemental sulfur initially dissolves and converts to high-order polysulfides (Li_2_S_8_) before following the same reduction pathway with identical voltage responses at 2.1 V. The effect of slightly different cycling rates (C/20 vs C/10) on the nanostructure is minor.^14^

The TEM grid was transferred under an Ar atmosphere from the glovebox to the TEM using a double tilt LN2 vacuum transfer holder (VTC, MelBuild) and a Gatan 648 double tilt vacuum holder. TEM measurements were carried out on a double Cs-corrected JEOL GrandARM operated at 300 kV (ETH Zürich) and at 80/200 kV on a double Cs-corrected JEOL JEM 2200 fs (Philipps-Universität Marburg). We followed two different LN2 cryovacuum-transfer-holder transfer procedures, depending on the microscope. For measurements on GrandARM, it works as follows: first, the holder loaded with the TEM-grid was transferred from the glovebox to the TEM. Once the sample was inside the airlock of the TEM, the chamber was purged three times with nitrogen. During the third pumping cycle, the tip was extended into the airlock at an ion pump current of 200 μA.

Alternatively, for measurements on a JEM 2200 fs, which is equipped with a diffusion instead of turbomolecular pump, the holder is first prepumped at a pumping a pumping station overnight. The tip is again extended during pumping. The next morning, the tip is retracted, and the holder is transferred to the microscope. In this case, the tip is extended into the airlock in the later stages of pumping. After the vacuum was stabilized, the holder is inserted into the column. In both cases, the holder was then cooled to −193 °C by introducing liquid nitrogen (LN2) into the holder’s dewar. To mitigate LN2 boiling and associated vibrations, helium gas was introduced into the cryo-container, further reducing the temperature to −198 °C, at which point the bubbling ceased.^49,50^ The system was allowed to equilibrate for one h, after which the LN2 was replenished and the helium gas cycle repeated. Following a second hour of stabilization, the system reached thermal equilibrium with a drift rate of approximately 1.5 nm/min, at which point data acquisition commenced. During the cryomeasurements, the tip was cooled to −165 °C to reduce drift.

### Machine Learning and SANS Data Fitting

All simulations and fitting procedures were performed on a high-performance Linux-based system equipped with a 16-core AMD Threadripper PRO 5955WX processor (4 GHz), 64 GB DDR4 3200 MHz ECC memory, and an NVIDIA RTX 4090 GPU with 24 GB of VRAM.

We implemented a custom CNN model using PyTorch to predict the SANS intensity curves from input parameters. The architecture comprises three blocks, each containing one deconvolutional layer and two convolutional layers with ReLU activation functions, followed by max pooling. The network takes 8 input parameters and outputs a 117-point intensity curve. Dropout layers (*p* = 0.5) were inserted throughout the blocks to mitigate overfitting. A flattening layer was employed to generate the final 1D output. The model’s trainable parameters totaled approximately 3 million. Adam with a learning rate of 8 × 10^–4^ and at StepLR scheduler with a step size of 4 and gamma of 0.5 was chosen.

A data set of 250,000 simulated small angle scattering (SAS) curves was generated based on the PGRF model. The parameter ranges used for the simulation are detailed in Table S1. This data set was partitioned into training (80%), validation (10%), and test (10%) subsets. Prior to training, we added the experimental background, applied logarithmic transformation to the intensity values, and normalized the intensity value and all parameters to the [0, 1] interval based on their respective global minima and maxima. Training was conducted over 40 epochs with consistent batch sizes of 256 for train, 128 for validation and test subsets. This process was repeated for four times. For the fitting process, the four networks were combined and their prediction averaged.

Experimental data fitting was performed using the Optuna library, implementing a Bayesian optimization algorithm with the mean squared error as the optimization metric. For handling the SANS intensity background at high *q*-values, we added the experimental background to the simulated PGRF curves during CNN training rather than subtracting it from experimental data (we selected the background SANS curve at the point where all sulfur was dissolved and no other solid phases were present; Figure 4c, potential drop before onset of the second discharge plateau). This approach proved more robust as it improved the CNN’s ability to recognize weak scattering features and enabled direct fitting to untreated scattering data (Figure S7). The experimental logarithmic intensity data underwent normalization based on the simulated data set’s minimum and maximum intensity values. Hence, all simulated SANS intensities are normalized between 0 and 1. This normalization approach is valid because our simulated data set was designed to span the largest possible range of scattering curves, ensuring that the experimental data to be fitted falls within the boundaries established by the training set’s intensity extremes. The optimization process evaluated 10,000 parameter combinations over a duration of 35 min.

### The PGRF SANS Model

In the following, we briefly describe the PGRF concept for calculating SANS intensities of a Li_2_S–Li_2_S_2_ composite nanostructure, in line with recent works.^14,40^Figure S5 summarizes the procedure. The experimental SANS intensity of the discharged cathode in absolute units (cm^–1^) can be decomposed into two main components
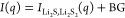
1 corresponds to the scattering from the Li_2_S/Li_2_S_2_ structure. BG accounts for the constant background intensity, primarily from incoherent scattering and the atomic structure factor of the electrolyte and carbon. Scattering from the carbon black nanostructure is negligibly small, as the deuterated electrolyte approximately matches the SLD of the carbon.

The experimental SANS intensity of the Li_2_S/Li_2_S_2_ nanostructure (in units of cm^–1^) can be written as
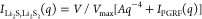
2*V*/*V*_max_ is the relative volume of the deposited Li_2_S/Li_2_S_2_ nanostructure and allows us to account for the changing deposit volume during cycling. *V*_max_ corresponds to the irradiated beam area (approximately 1 cm in diameter) times an effective Li_2_S/Li_2_S_2_ deposit thickness *d*_max_. We chose 0.007 cm as an initial estimate for *d*_max_ and used this value for the normalization to absolute intensities. The first term, *Aq*^–4^, describes the Porod decay from large Li_2_S/Li_2_S_2_ agglomerates (larger than 100 nm, see Figures 3a and S1), with the SANS intensity being proportional to *q*^–4^ within the measured *q*-range. The second term, *I*_PGRF_(*q*), models the nanostructure within the 1–50 nm range by using PGRF.

The SANS intensity *I*_PGRF_(*q*) can be represented as the Fourier transform of the SLD correlation function *C*(*r*). This relationship is given by
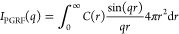
3

For the three-phase system composed of Li_2_S, Li_2_S_2_, and electrolyte, *C*(*r*) can be expressed as
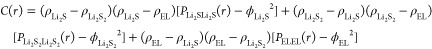
4where ρ_i_ denotes the scattering length density, ϕ_i_ the volume fraction, and *P*_ii_(*r*) the two-point correlation function of phase i.

By employing GRF, one can create a 3D model of a two-phase pore structure from the fit to an experimental scattering curve. PGRF extend this concept by combining two GRFs to model SANS intensities and 3D structures in three-phase systems. A GRF *Y*(**x**) is defined as
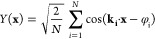
5
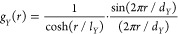
6where *l*_*Y*_ is a correlation length parameter related to the feature size of the structure and *d*_*Y*_ characterizes ordering effects.

The power spectral density associated with *g*_*Y*_(*r*) can be written as
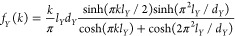
7

To generate a two-phase porous structure from the GRF, the threshold value α is introduced. All points ***x*** for which α < *Y*(***x***) ≤ ∞ are assigned to the pore space (the combined Li_2_S_2_ + EL phase), while other coordinates correspond to the Li_2_S scaffold. The threshold α is linked to the Li_2_S volume fraction  by
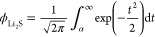
8

For the three-phase system, a second independent GRF *Z*(*x*) is generated sharing the same functional form of the correlation function but distinct parameters *l*_*Z*_ and *d*_*Z*_. The Li_2_S_2_ phase is obtained by combining *Z*(*x*) and *Y*(***x***) and applying thresholds in the *Y*,*Z* partition plane (Figure S5)
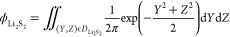
9

The two-point correlation function for the Li_2_S_2_ phase is
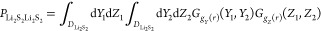
10

These distributions are obtained using Hermite polynomials^40^ (similar considerations apply for Li_2_S). The corresponding scattering intensities can then be calculated from correlation functions using eqs 3 and 4. The morphology of the Li_2_S_2_ phase is influenced by the angle β and the Li_2_S_2_/EL boundary line, as shown in Figure S5. When β → 0, the Li_2_S_2_ phase forms a thin film perfectly coating the Li_2_S phase. Conversely, when the Li_2_S_2_/EL boundary is parallel to the *Y*-axis (β → π/2), the Li_2_S_2_ (or EL) structure within the Li_2_S pores becomes statistically independent of the Li_2_S structure.

## Data Availability

The Python implementation of the PGRF algorithm and the machine learning models are openly available on GitHub (https://github.com/JeanvonMentlen/machine_learning_enhanced_pgrf). All raw experimental data and the PGRF data set used to train the CNN have been deposited in Zenodo and are accessible at DOI: 10.5281/zenodo.14532384.

## Supporting Information Available

Further micrographs and raw EELS spectra; impact of the solvent used to wash samples; PGRFs concept and the impact of parameters on the structure; data pipelines and training results for the forward model and inverse problem; input parameter range of the PGRF data set; and SLDs for the materials used (PDF)

## Notes

Preprint Jean-Marc von Mentlen, Ayça Senol Güngör, Thomas Demuth, Jürgen Belz, Milivoj Plodinec, Pronoy Dutta, Alen Vizintin, Lionel Porcar, Kerstin Volz, Vanessa Wood, Christian Prehal, Exploring multiphase conversion pathways in Li–S batteries through cryo-TEM and ML-assisted operando neutron scattering, 23 December 2024, PREPRINT (Version 1), Research Square 10.21203/rs.3.rs-5689820/v1 (accessed April 15 2025).
