# Supplementary material for: Unraveling Multiphase Conversion Pathways in Lithium–Sulfur Batteries through Cryo Transmission Electron Microscopy and Machine Learning-Assisted Operando Neutron Scattering
Source: ACS Nano. 2025 Apr 24;19(17):16626–38. doi: 10.1021/acsnano.5c00536 (PMC12060645; doi:10.1021/acsnano.5c00536)
Supplement: Supplementary file 1 — nn5c00536_si_001.pdf [file nn5c00536_si_001.pdf]

Supporting Information

Unraveling Multiphase Conversion Pathways in Lithium-Sulfur Batteries through Cryo-Transmission Electron Microscopy and Machine Learning Assisted *Operando* Neutron Scattering

Jean-Marc von Mentlen[1], Ayça Senol Güngör[1], Thomas Demuth[2], Jürgen Belz[2], Milivoj Plodinec[3], Pronoy Dutta[4], Alen Vizintin[5], Lionel Porcar[6], Kerstin Volz[2], Vanessa Wood[1,*], Christian Prehal[1,4,**]

1 Department of Information Technology and Electrical Engineering, ETH Zürich, Gloriastrasse 35, 8092 Zürich, Switzerland

2 Materials Science Center and Faculty of Physics, Philipps University Marburg, Hans-Meerweinstraße 6, Marburg 35043, Germany

3 Scientific Center for Optical and Electron Microscopy, Department of Chemistry and Applied Biosciences, ETH-Zürich, Otto-Stern-Weg 3, 8093 Zürich, Switzerland

4 Department of Chemistry and Physics of Materials, University of Salzburg, Jakob-Haringer-Strasse 2a, 5020 Salzburg, Austria

5 Department of Materials Chemistry, National Institute of Chemistry, Hajdrihova 19, 1000 Ljubljana, Slovenia

6 Institut Laue–Langevin, 71 Avenue des Martyrs, Grenoble, 38042, France

*Correspondence: vwood@ethz.ch
**Correspondence: christian.prehal@plus.ac.at

TEM Data

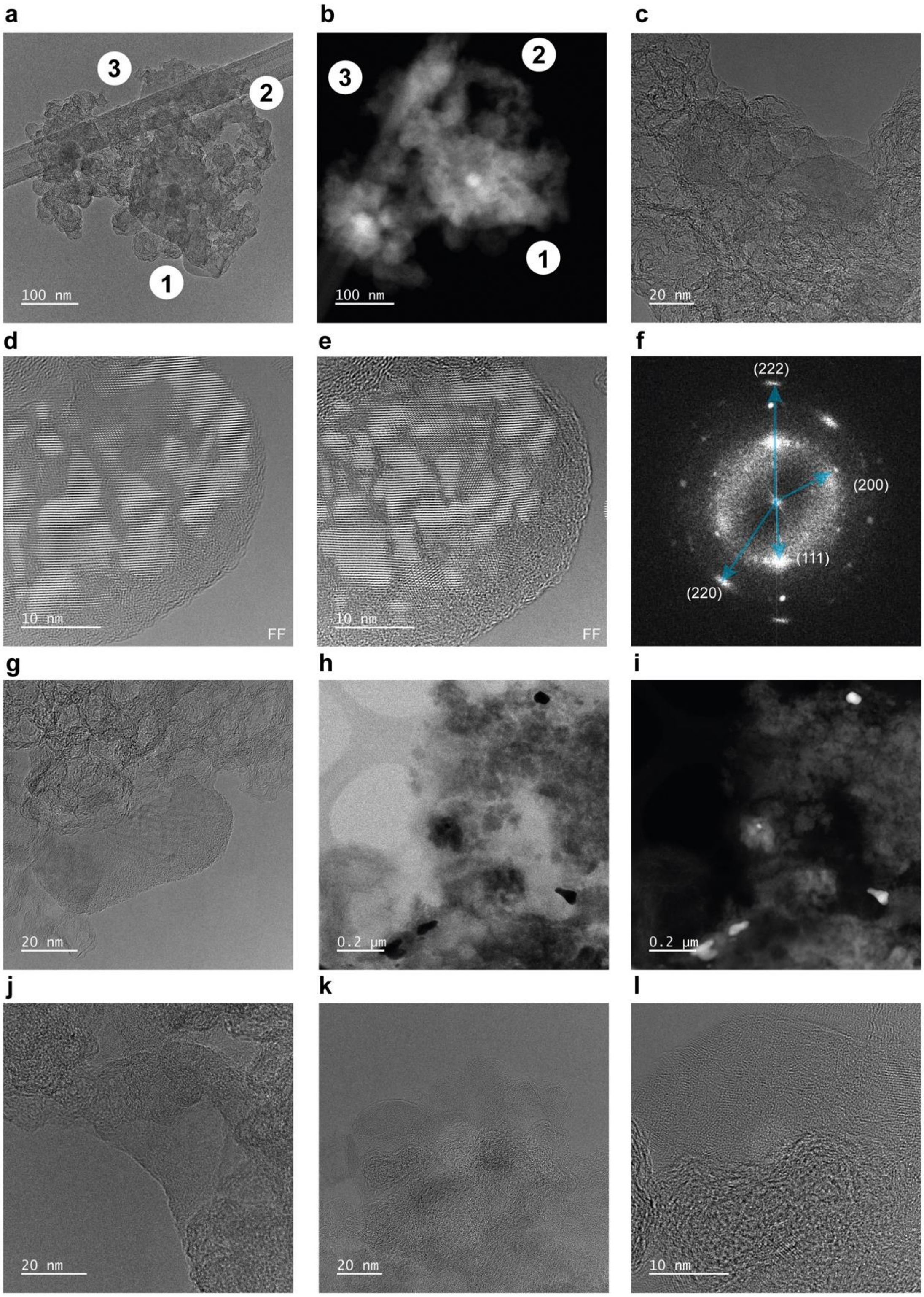

Supporting Figure 1 ***TEM and STEM micrographs.*** *CryoTEM a-c): a) A typical cathode particle with Ketjen Black (KB) carbon and discharge product (DP) labelled 1-3 in HRTEM. b) Contrast between KB and DP low and dominated by sample thickness. c) Magnified view on DP. Ambient-TEM d-g): d) The DP remained stable at ambient temperature. d,e) Magnified view at two different defocus values highlighting the complex, three-dimensional, two-phased structure, highlighted by Fourier filtering. f) FFT of d) showing spots associated to the (111), (200), (220), and (222) planes in* Li_2S*. CryoSTEM h,i) Brightfield and anular dark field (AFD) micrograph highlighting the strong contrast difference between salt crystals and the KB/DP structure. j-l) additional discharge particles under cryo (j) and ambient conditions (k, l),*

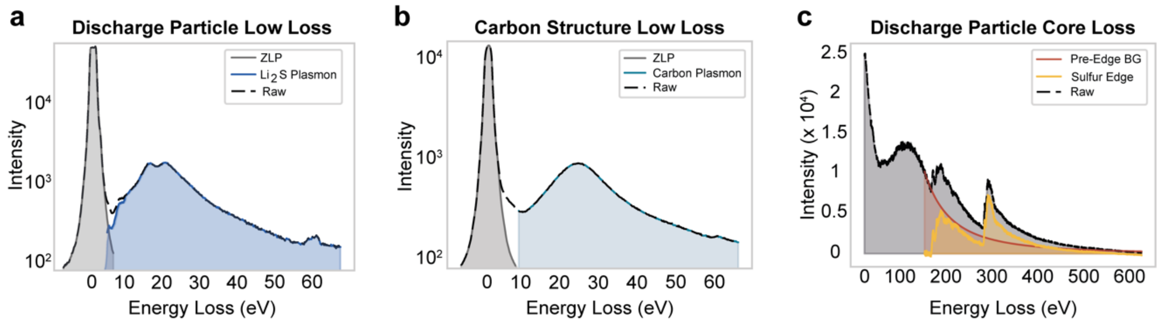

Supporting Figure 2 ***Full TEM-EELS.*** *The low loss spectrum of both a) the carbon structure and b) the discharge particle are not affected by the isolation and subtraction of the zero loss peak (ZLP), indicating that the particle is sufficiently thin. c) Core loss spectrum with a power law background subtracted.*

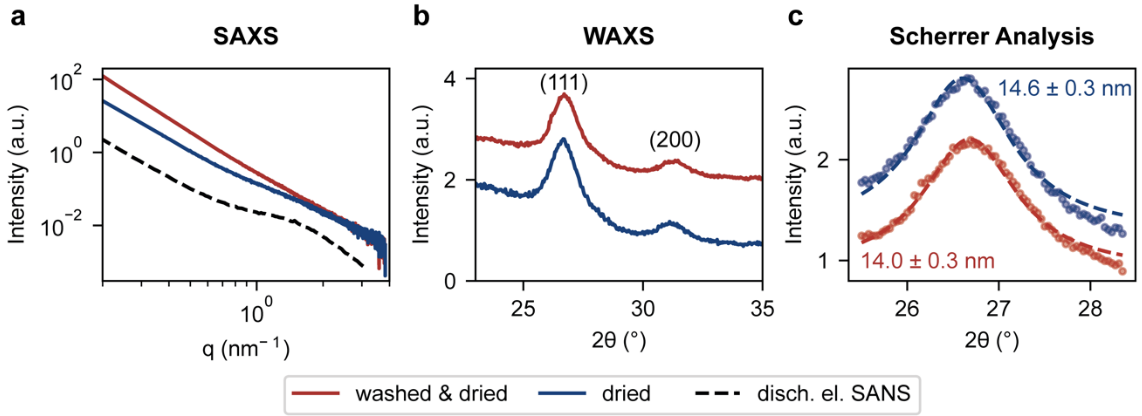

Supporting Figure 3 ***Washing experiment to clarify the origin of the high-q SANS shoulder.*** *SAXS/SANS (a) and WAXS (b) intensities of discharge products and their solubility. The SANS curve of the discharged cathode in panel a (dashed black line, from the operando SANS data at 100 % SOD) shows a distinct shoulder at q ≈ 1.5 nm^{-1}. The SAXS curve of the dried discharged cathode shows the same shoulder (blue solid line in panel a). The (111) and (200) Li_2S diffraction peaks at $2\theta \approx 27.1°$ and 31.5° in WAXS confirm the existence of nanocrystalline Li_2S (blue solid line in panel b). Washing the discharged cathode with diglyme removes the SAXS shoulder while preserving the Li_2S peak (red), indicating that the high-q shoulder is caused by a partially soluble polysulfide (Li_2S_x) and not by insoluble Li_2S. c) Scherrer Analysis on the (111) Li_2S peak yielded crystallite sizes of 14.0 nm and 14.6 nm for the washed & dried and dried samples, respectively.*

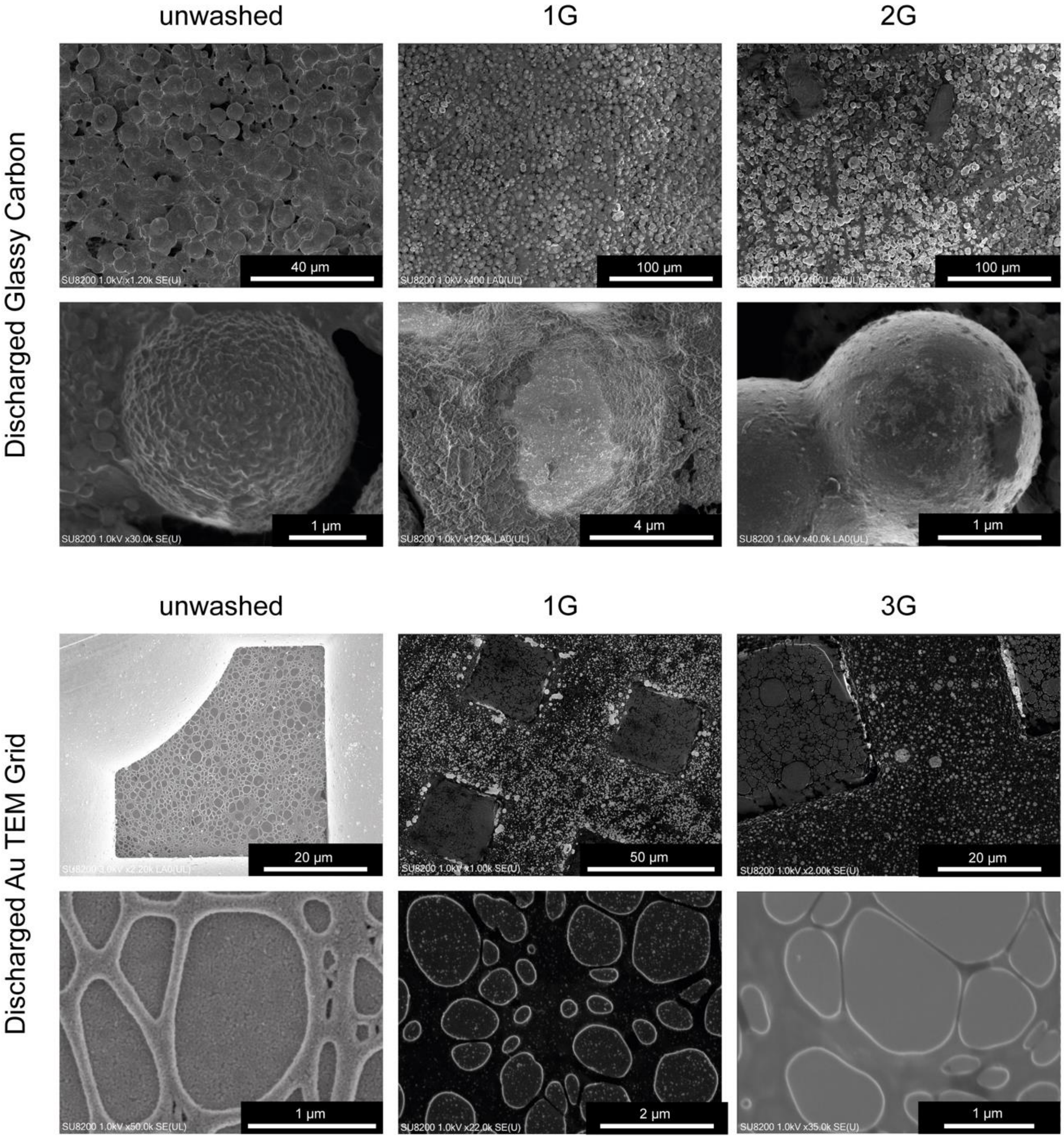

Supporting Figure 4 ***SEM micrographs showing the effect of different washing solvents on discharged cathodes.*** *Each row presents an overview (top) and detailed (bottom) view. Unwashed samples exhibit a continuous particle film covering the surface. Washing with monoglyme (1G) alters and partially removes this structure. In discharged TEM grids, large salt precipitates (white crystals) are visible in the overview, with finer particles in the detailed images. Washing with diglyme (2G) significantly removes the surface layer from glassy carbon, while triglyme (3G) almost completely eliminates visible structures, leaving only some bright residues in the overview.*

Plurigaussian Random Fields

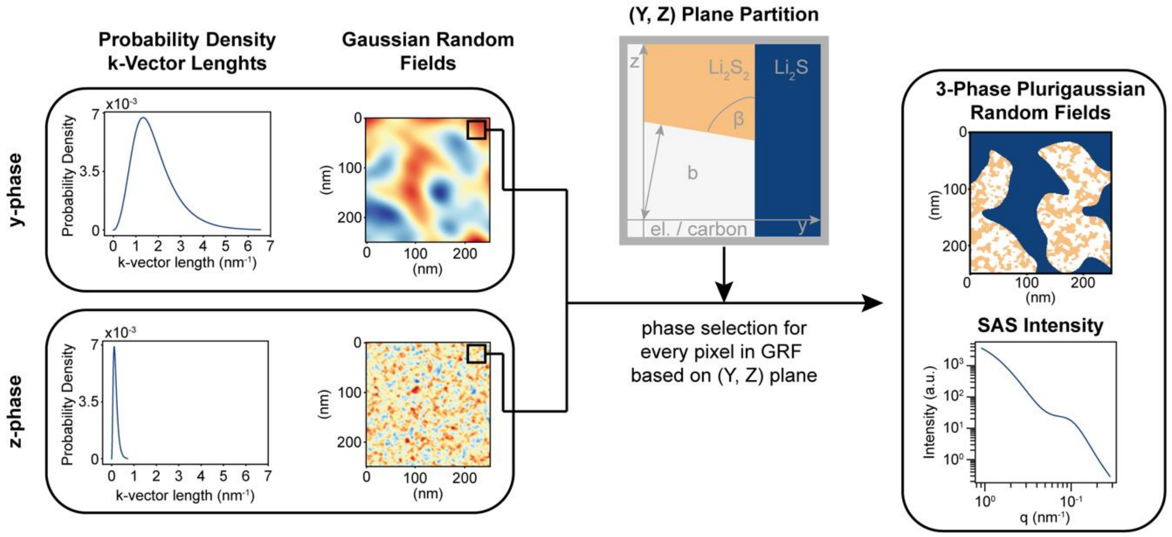

Supporting Figure 5 ***The stochastic Plurigaussian Random Field model.*** *The PGRF method generates two Gaussian random fields (GRFs) based on the probability density functions (PDFs) of k-vector lengths corresponding to the GRF, as defined by Equation 7 in the main text. Each PDF is characterized by input parameters (l_Y, d_Y) and l_Z, d_Z respectively) and thus reflects the feature sizes and structure of the y- and z-phase. The final structure is determined by comparing the intensity values of corresponding pixels in both GRFs against a (Y, Z) partitioned plane. This plane is defined by additional input parameters including b, beta and the phase fractions of the three phases. The PGRF function directly yields both small-angle scattering (SAS) intensity curves and their corresponding stochastically representative 3D structures.*

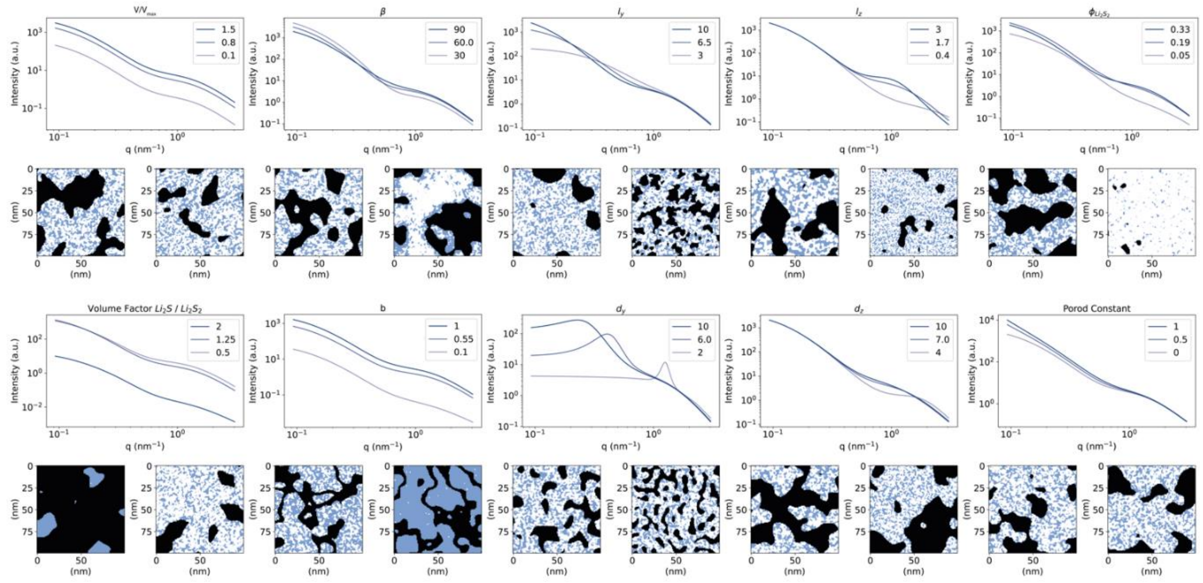

Supporting Figure 6 ***Parameter impact study.*** *A selection of three values for each of the ten PGRF input parameters with the impact on the SANS curve and the simulated three-phase electrode structure. The values represent the limits and the center value of the parameter range of the dataset. The left structure corresponds to the upper and the left structure to the lower limit.*

Forward Convolutional Neural Network Approach

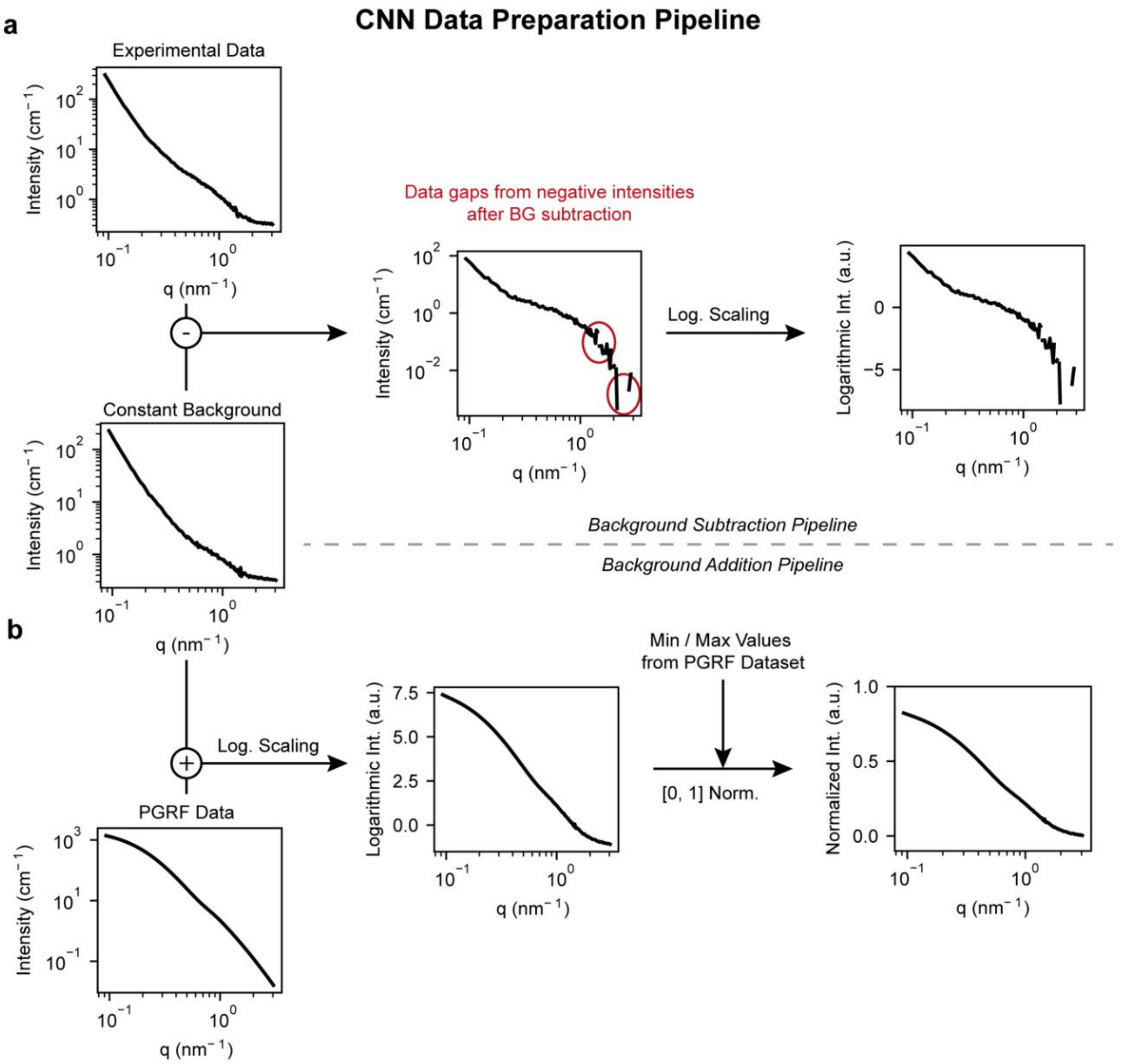

Supporting Figure 7 ***CNN Data Preparation Pipeline.*** *a) Background Subtraction Pipeline b) Background Addition Pipeline.*

Training the CNN benefits from normalizing SANS intensities between 0 and 1, which is best practice in machine learning as this improves training stability and convergence.

Background subtraction approach: subtract background from experimental SANS curves, normalize BG subtracted logarithmic experimental SANS intensities to values between 0 and 1 and train CNN on pure PGRF curves, fit to background-subtracted data. This method had two key limitations. First, the CNN struggled to learn parameter combinations that produced weak scattering intensities (specifically at high q). Second, during fitting, background subtraction in noisy high-q regions produced inconsistent negative values, which makes a problem when taking the logarithm of the intensity values. This required masking some data points, leading to poor optimization performance. This required masking some data points, leading to poor optimization performance.

Background addition approach (our chosen method): normalize logarithmic experimental SANS intensities to values between 0 and 1 and add background to simulated PGRF curves for training, train CNN to recognize background as part of the signal, fit directly to raw SANS

data.This method proved more robust because the added background improved CNN training for weak scattering features and avoided any manipulation of experimental curves, enabling direct fitting to untreated scattering data.

Based on these results, we implemented the background addition approach as it provided more reliable and consistent results.

8 variable – 2 fixed PGRF parameters with Li_2S, Li_2S_2 and Carbon for SLD parameters

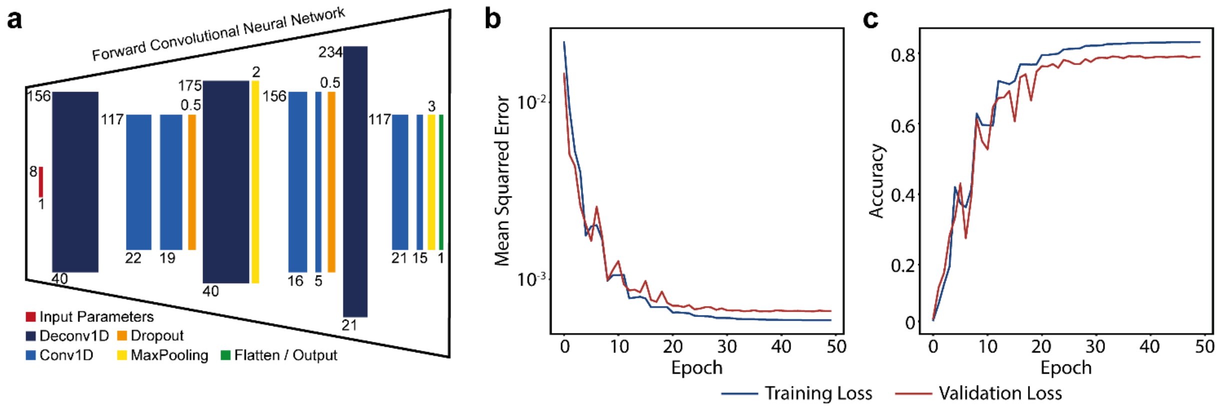

Supporting Figure 8 ***ForwardCNN model and training performance.*** *a) Architecture of the ForwardCNN with the layer size indicated for every block. b) Mean Squared Error (MSE) and c) accuracy training evolution. The accuracy parameter is defined by* $|target - output| < 0.005$.

Analysis of Outlier Curves

There are four sets of parameter – intensity pairs for which the model shows a significant larger error compared to the other curves (Fig. S5). In the following figures, we explore the parameters, and where they are positioned within the parameter range (Fig. S6) and show the predictions of the best (Fig. S7) and worst (Fig. S8) four curves vs. the targets.

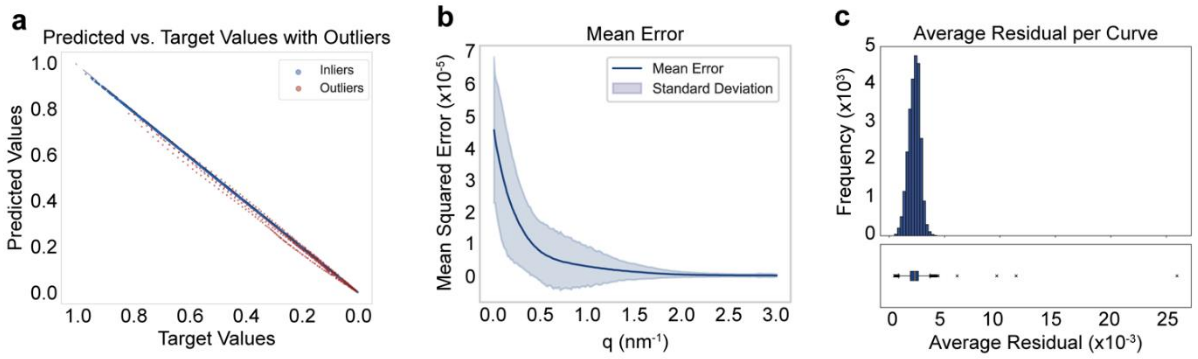

Supporting Figure 9 ***ForwardCNN performance analysis****. a) ForwardCNN prediction results on test dataset. The four curves with the worst prediction are highlighted in red. b) Mean squared error distribution with respect to the scattering vector. While the general MSE error is low (<10*$^{-5}$*), the model performs worse in the q-range below 1 nm*$^{-1}$*. c) Average residual (target-prediction) of each predicted intensity curve in the test dataset. With the exception of four outliers, predicted curves deviate less than 0.5% from the target values.*

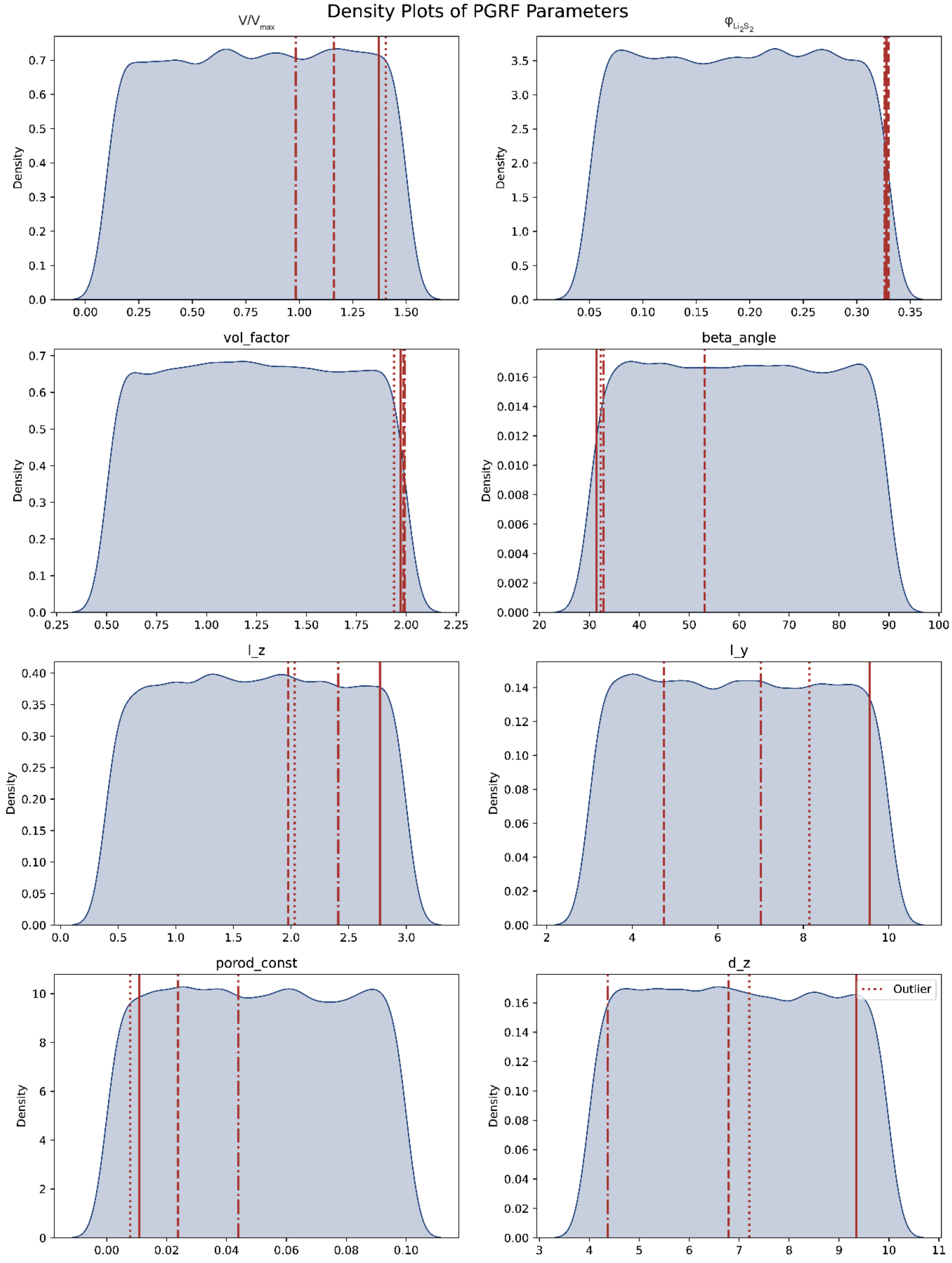

Supporting Figure 10 ***Parameter distribution of outlier curves.*** *The distribution of all eight parameters (blue) is shown with the values of the parameters corresponding to the four outlier curves (red) overlayed.*

The parameters phi_Li_2S_2, beta, and volume_factor for the outlier curves are located at the edge of the parameter distribution, which signifies edge cases of certain structures. For example, a volume fraction of the Li_2S_2 phase (phi_Li_2S_2) of 0.33 paired with a volume factor of 2 means that the Li_2S_2 phase (Li_2S_2) and Li_2S phase make up 99% of the three phases in the structure. Small beta values, as another example, can lead to issues with the numerical integration. We, therefore, set the lower limit to 30°. However, the neural network struggles to learn the relationship between parameters and intensity curves in the case of combining several parameters at the limit of their range.

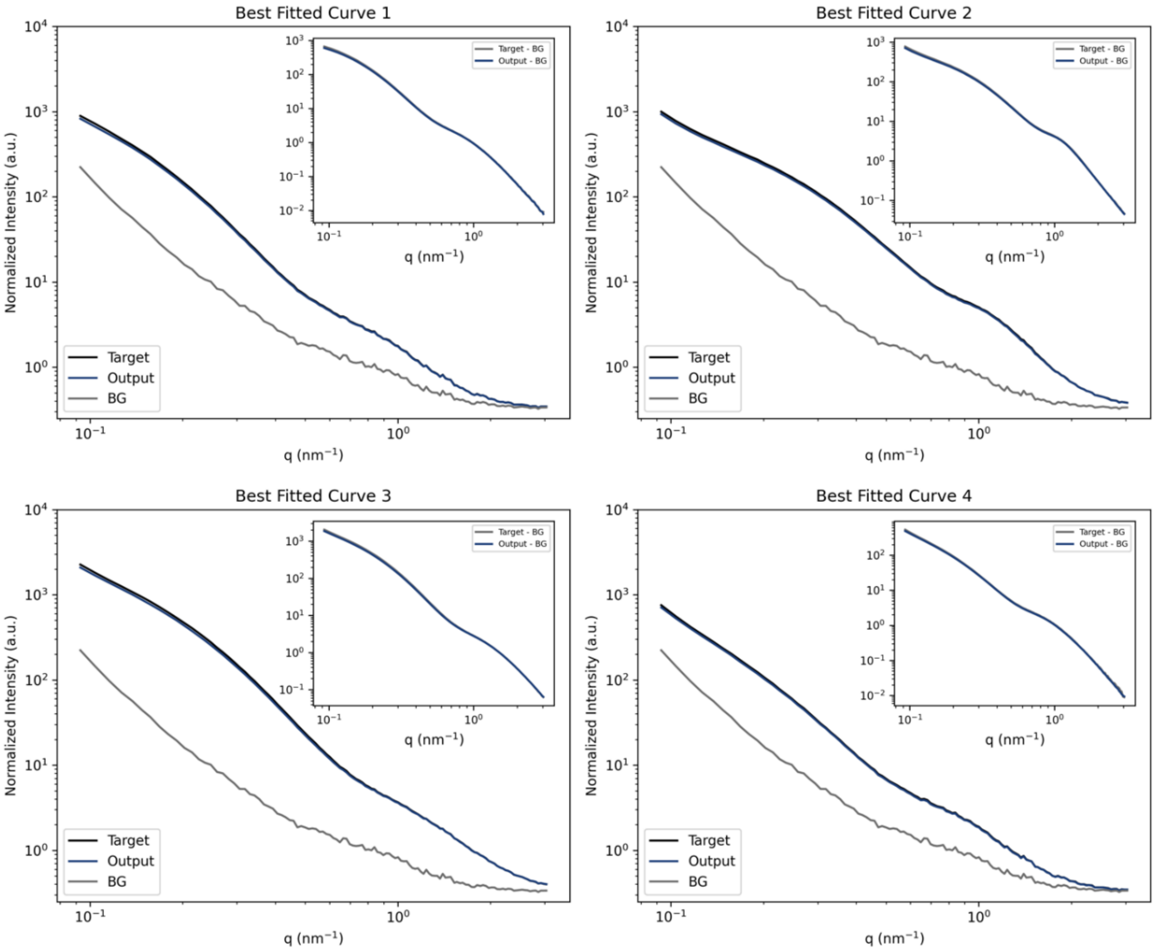

Supporting Figure 11 ***Performance excerpt – best predictions.*** *Showcase of the four intensity curve from the test dataset that were predicted best. Target (blue) and the ForwardCNN output (blue) are compared with each other with (large plot) and without (inset plot) experimental background.*

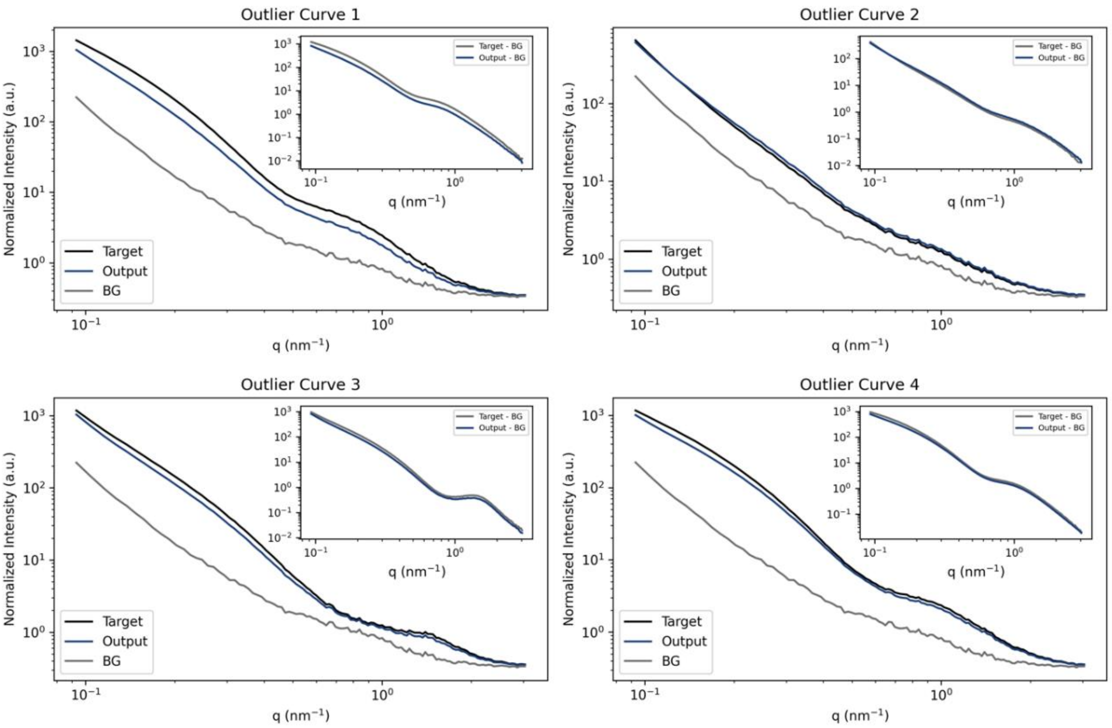

Supporting Figure 12 ***Performance excerpt – worst predictions.*** *Showcase of the four intensity curve from the test dataset that were predicted worst and were highlightet in S5. Target (blue) and the ForwardCNN output (blue) are compared with each other with (large plot) and without (inset plot) experimental background.*

8 variable – 2 fixed PGRF parameters with variable scattering length density (SLD) parameters

In the scope of this project, the phases of our systems were well-determined, allowing us to fix the SLD during the dataset generation for our model training. However, to generalize the approach for broader applications, we also created a more comprehensive dataset of 700,000 PGRF parameter and intensity curve pairs. We expanded the parameter range of the SLD to cover the most common conversion-type batteries, setting it to $\boldsymbol{\rho_{A,S}} \in [-\mathbf{0.3}, \mathbf{4}] \times \mathbf{10^{10}}\ \boldsymbol{cm^{-2}}$ for the active materials and $\boldsymbol{\rho_B} \in [\mathbf{4}, \mathbf{7}] \times \mathbf{10^{10}}\ \boldsymbol{cm^{-2}}$ for conductive additives and solvents such as carbon.

Initial tests with the ForwardCNN model on this expanded dataset revealed both challenges and promising results. Due to the increased dataset size and the architecture used in our previous approach, the model showed a tendency to overfit on the training data. Nevertheless, the resulting performance plot confirmed that the model could still predict this unrestricted dataset with high precision, demonstrating the potential of this generalized approach.

Based on these initial findings, we propose two pathways for further research:

1. Utilize the existing ForwardCNN model architecture with the pretrained model in the Bayesian optimization loop. When the chemical composition of the sample is known, set the SLD constant to the known values. For unknown compositions, provide a list of fixed SLD values for potential materials to be used in the optimization loop. This approach would allow the system to suggest the material that leads to the lowest fitting error.

2. Use the provided dataset as a foundation for developing new models with different architectures. This would involve adapting or rewriting the optimization script to accommodate the new model structure.

Given that data generation is the most time-consuming aspect of this process, we believe our expanded dataset provides a valuable starting point for further exploration and refinement of the methods presented here. By making this resource available, we hope to facilitate continued research and improvement in SANS data analysis techniques for a wider range of material systems.

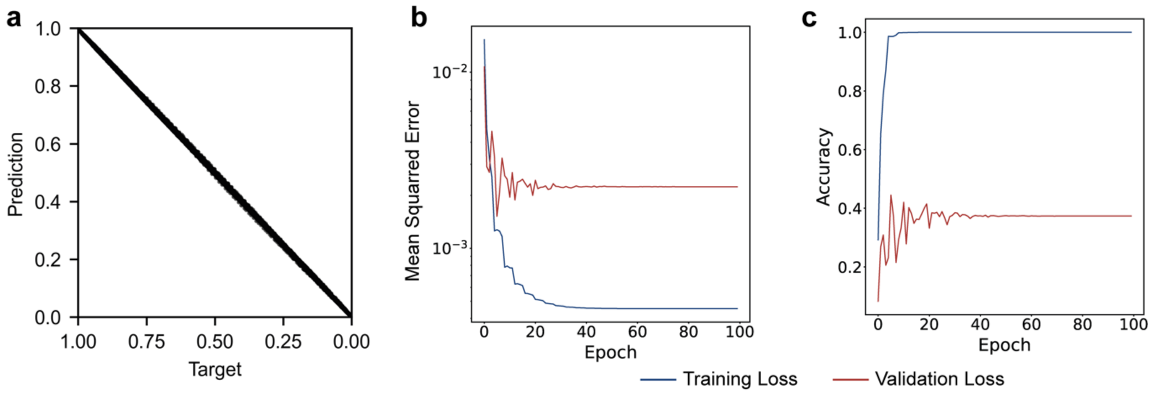

Supporting Figure 13 ***Training results for ForwardCNN on 8 PGRF and 3 SLD parameters.*** *a) Performance prediction b) training error and c) accuracy evolution.*

Inverse Convolutional Neural Network Approach

In our exploration of efficient SANS data analysis methods, we investigated an inverse fitting approach using a convolutional neural network (InverseCNN). This method aimed to predict PGRF parameters directly from SANS intensity curves, potentially bypassing the computationally intensive Bayesian optimization algorithm used in our primary approach.

The InverseCNN's architecture (Figure S10a) closely resembles that of the ForwardCNN, differing mainly in layer sizes. However, a key distinction lies in the training process: for each set of variables to be predicted, a new dataset with specific free and constant parameters must be generated, and the model retrained. This requirement introduces a significant limitation in the model's flexibility and adaptability.

Our comparative analysis of two InverseCNN models, trained to predict four and eight PGRF parameters respectively (Figure S10d), revealed two crucial insights. First, the model's performance deteriorates with an increasing number of free parameters. Second, certain parameters prove more challenging to predict accurately. This variability in prediction accuracy can be attributed to the similar impacts some parameters have on the scattering curve, as demonstrated in our parameter impact study (Figure S4).

We tested the performance of the InverseCNN with four variable parameters on experimental data by using its predicted PGRF parameters as inputs for the analytical PGRF function. The resulting SANS intensity curves, overlaid with experimental data in Figure S11 a, b, show mixed results. While the InverseCNN adequately predicts parameters for the discharge cycle, it struggles to describe the hump at 0.8 nm^{-1} and fails entirely for data near the end of charge.

To better understand these discrepancies, we compared the InverseCNN's predictions with those from our ForwardCNN + Bayesian optimization approach. Figure S11d-I illustrates this comparison, including two InverseCNN models (trained on four and eight variable parameters) and the ForwardCNN predicting six parameters. The forward approach consistently outperforms the inverse models, with a mean squared error (MSE) two to three orders of magnitude smaller.

A fundamental issue with the inverse approach is its tendency to "hallucinate" - predicting values outside the training data range. This problem, common in many neural networks, is particularly concerning for scientific applications where accuracy is paramount. Our forward approach mitigates this issue through the Bayesian optimization loop and manual setting of parameter ranges during fitting.

In conclusion, while the inverse approach shows potential, it faces significant challenges that led us to favor the forward approach. The need for specifically trained models for each parameter set limits flexibility, and the addition of more variables (such as scattering length densities) would likely further reduce performance. The difficulty in addressing the hallucination problem also undermines confidence in the model's predictions.

Although we remain open to the possibility of a successful inverse approach, our research suggests that more sophisticated architectures would be necessary to achieve the required

accuracy and flexibility. For now, our forward approach offers a more reliable and adaptable solution for SANS data analysis, effectively balancing performance and trustworthiness.

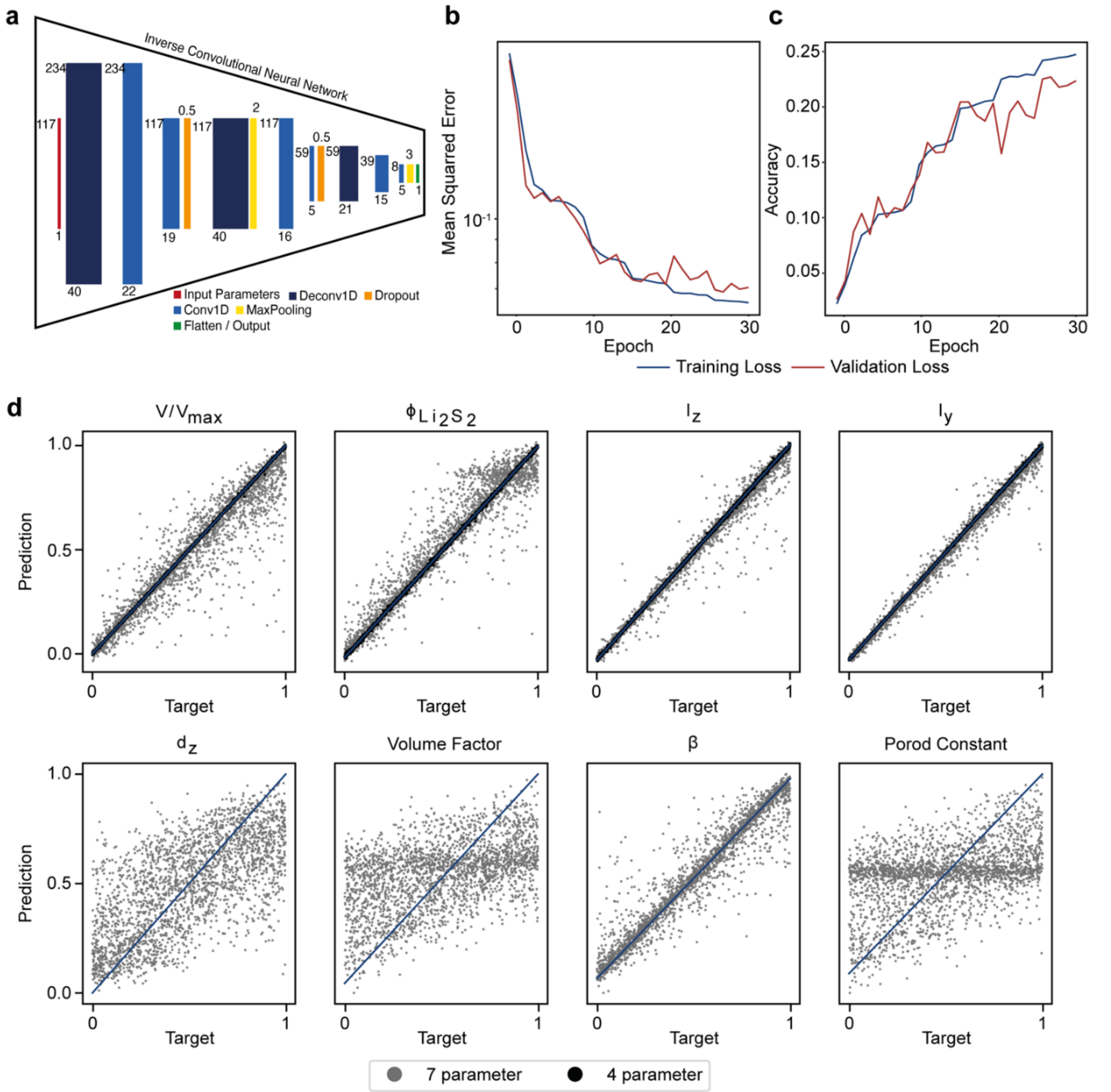

Supporting Figure 14 ***InverseCNN Model – Predict the PGRF directly from the SANS intensity****. a) Architecture of the InverseCNN with the layer size indicated for every block. Evolution of the training metrics b) Mean Squared Error (MSE) and c) Accuracy. d) Prediction results on test dataset of two InverseCNN models, one trained to predict four (black) and eight (grey) PGRF parameter.*

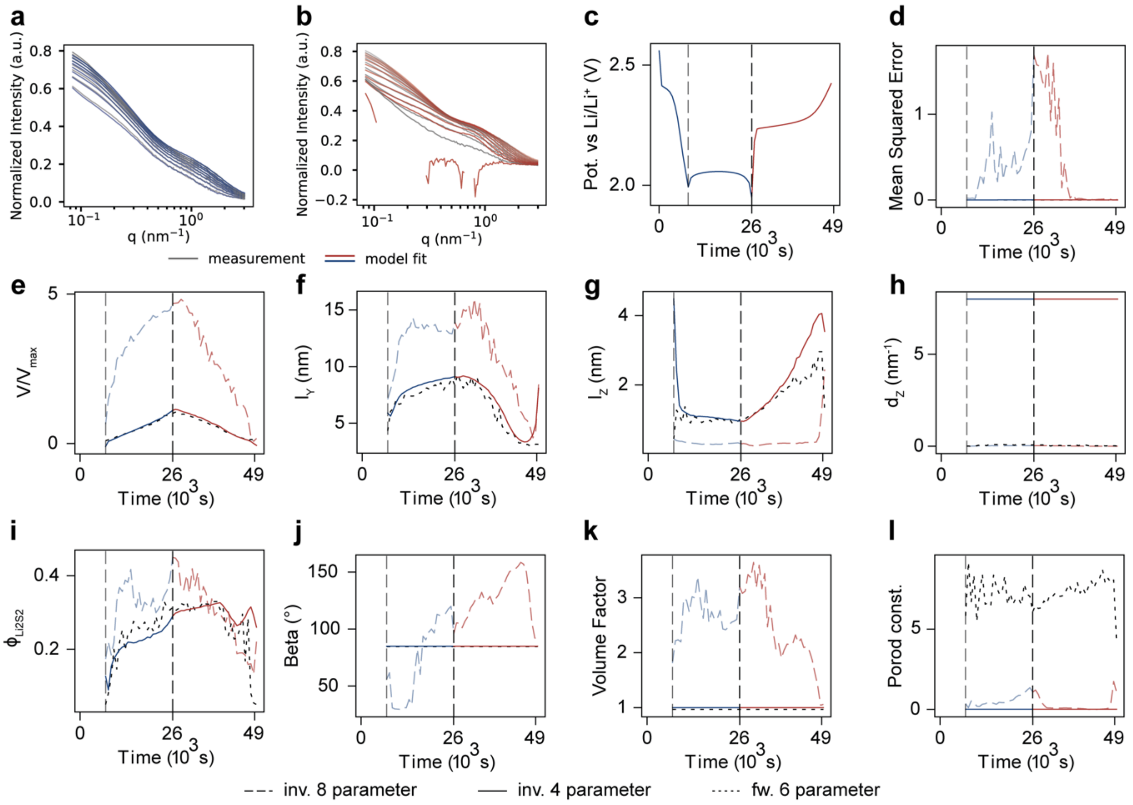

Supporting Figure 15 ***InverseCNN Model – Predict the PGRF directly from the SANS intensity****. Predicted curves for a) discharge (blue) and b) charge (red) with the experimental curves (grey). c) discharge (blue) and charge(red) profile of operando SANS cell. d) Mean squared error of normalized fitted vs target curves. e-l) The predicted PGRF parameters for all experimental curves for eight variable (dashed) and four variable (solid) parameters are compared to fitted parameters using a ForwardCNN with six variables.*

Simulation of Training Data

Intensity curves from small angles were derived using a Python-based adaptation of the Plurigaussian Random Fields (PGRF) as described in the work by Gommes[1]. The source code for this implementation is publicly accessible[2]. Analytical computations of the intensities were performed using q-vector values sourced from the SANS experiment, along with a randomized selection of PGRF input parameters. The span of these parameters is detailed in Table 1. From this process, we created a dataset with 400,000 intensity curves.

Supporting Table 1: PGRF parameter range. The data set to train the CNN was generated using the above parameter range.

Variable Parameter	Maximum Value	Minimal Value
V/V_{max} ('int_const')	1.5	0.1
$\boldsymbol{\phi}_{Li_2S_2}$ ('phi_A')	0.33	0.05
volume_factor	2	0.5
beta_angle	90	30
l_z	3	0.4
l_y	10	3.5
d_z	10	4
Constant Parameter	Value	
d_y	150	
porod_const	0	
b	5.8	

Variable SLD Dataset

rho_A	-0.3	4
rho_B	4	7
rho_S	-0.3	4

Supporting Table 2: Scattering length densities (SLDs) of the various phases. The Li_2S_2 mass density, 1.62 cm^3 g^{-1}, was taken from DFT simulations, calculating crystal structures of solid PSs[3]. For the catholyte, we assumed a Li_2S_8 concentration, higher than in the bulk liquid, resulting in an SLD of $1.1x10^{11}$ cm^{-2} for SAXS. For the SANS model fit we assumed that the electrolyte (1 M LiTFSI + 0.4 M LiNO3 in 2G) SLD matches the carbon SLD. The carbon black skeleton density is lower than the graphite density due to the significant micropore content[4,5].

	Mass density ($g\ cm^{-3}$)	Molar mass ($g\ mol^{-1}$)	Molar volume ($cm^3\ mol^{-1}$)	SLD_{SANS} (cm^{-2})
Li_2S	1.66	45.95	27.68	-0.21×10^{10}
Li_2S_2	1.62	78	48.15	0.24×10^{10}
S	2.00	32	16	1.11×10^{10}
2G (deuterated)	-	-	-	6.34×10^{10}
Carbon	2.0	12	5.85	6.67×10^{10}
Electrolyte (deuterated)	-	-	-	5.63×10^{10}

References

1. Gommes, C. J. Three-dimensional reconstruction of liquid phases in disordered mesopores using in situ small-angle scattering. *J Appl Crystallogr* **46**, 493–504 (2013).
2. von Mentlen, J.-M. Machine Learning Enhanced PGRF Simulation. Preprint at https://github.com/JeanvonMentlen/machine_learning_enhanced_pgrf (2024).
3. Xiao, J. *et al.* Elaboration of Aggregated Polysulfide Phases: From Molecules to Large Clusters and Solid Phases. *Nano Lett* **19**, 7487–7493 (2019).
4. Bahadur, J. *et al.* SANS investigations of CO2 adsorption in microporous carbon. *Carbon* **95**, 535–544 (2015).
5. Prehal, C. *et al.* A carbon nanopore model to quantify structure and kinetics of ion electrosorption with in situ small-angle X-ray scattering. *Physical Chemistry Chemical Physics* **19**, 15549–15561 (2017).
